# Decision prioritization and causal reasoning in decision hierarchies

**DOI:** 10.1371/journal.pcbi.1009688

**Published:** 2021-12-31

**Authors:** Ariel Zylberberg

**Affiliations:** 1 Zuckerman Mind Brain Behavior Institute, Columbia University, New York, New York, United States of America; 2 Department of Brain and Cognitive Sciences, University of Rochester, Rochester, New York, United States of America; Chinese Academy of Sciences, CHINA

## Abstract

From cooking a meal to finding a route to a destination, many real life decisions can be decomposed into a hierarchy of sub-decisions. In a hierarchy, choosing which decision to think about requires planning over a potentially vast space of possible decision sequences. To gain insight into how people decide what to decide on, we studied a novel task that combines perceptual decision making, active sensing and hierarchical and counterfactual reasoning. Human participants had to find a target hidden at the lowest level of a decision tree. They could solicit information from the different nodes of the decision tree to gather noisy evidence about the target’s location. Feedback was given only after errors at the leaf nodes and provided ambiguous evidence about the cause of the error. Despite the complexity of task (with 10^7^ latent states) participants were able to plan efficiently in the task. A computational model of this process identified a small number of heuristics of low computational complexity that accounted for human behavior. These heuristics include making categorical decisions at the branching points of the decision tree rather than carrying forward entire probability distributions, discarding sensory evidence deemed unreliable to make a choice, and using choice confidence to infer the cause of the error after an initial plan failed. Plans based on probabilistic inference or myopic sampling norms could not capture participants’ behavior. Our results show that it is possible to identify hallmarks of heuristic planning with sensing in human behavior and that the use of tasks of intermediate complexity helps identify the rules underlying human ability to reason over decision hierarchies.

## Author summary

Complex decisions are often broken down into a sequence of information-gathering actions followed by reward-seeking actions. For example, a physician may conduct a series of tests to diagnose a patient’s disease before suggesting a corrective action. How do people decide what is the appropriate question (test, experiment, query) to ask next? Human participants were presented with a binary decision tree that bifurcated three times. They could solicit information from the bifurcation points to gather noisy evidence about the location of a target. We identified the heuristics that people used to plan efficiently in this complex task. Participants exploited the hierarchical structure of the task and relied on the confidence in past decision to inform the selection of subsequent actions. Our results bear on how people plan efficiently in large partially observable domains, and have implications for the design of artificial agents that have to make decisions with active exploration and for neurophysiological studies of planning in humans and other animals.

## Introduction

Many real-life decisions are organized hierarchically in the sense that they are composed of parts that themselves can be considered decisions. As an example, consider an engineer who must diagnose the cause of failure at an industrial plant. The engineer might break down this complex decision into a sequence of simpler ones (e.g., did the motor trip? If so, did a fuse blow? If not, did the pump bearings fail?). These decisions are resolved by specific information-seeking actions (i.e., tests), where the outcome of each test influences the subsequent ones. Information-seeking actions are usually followed by reward-seeking ones. For example, the engineer may conclude that the failure was due to wear of the pump bearings and decide to replace them. Often, our actions do not lead to the desired outcome and we need to replan [1–3]. For instance, if changing the pump’s bearings does not restore the plant’s function, the engineer must determine the cause of her flawed reasoning and decide what action to take next (e.g., conduct a new test, repeat an unreliable one, or replace a different component).

The example is representative of many decisions that comprise a hierarchy of sub-decisions and where feedback does not allow the cause of errors to be unambiguously identified. They appear in medical diagnoses, when choosing a career path or designing an experiment to address a scientific question. These decisions are difficult to make optimally for three reasons. First, the decision maker must choose which actions are most relevant at each step, which is complex because the number of possible action sequences grows exponentially with the planning horizon and the value of an action may depend on the entire sequence of past actions and observations [4, 5]. For example, the relevance of inspecting the pump bearings for wear depends on whether a previous test pointed to the pumping system as a probable cause of failure. Second, some information-seeking actions may be more reliable or costly than others, which should inform the selection of the next action [6, 7]. For example, if the engineer learns that a test is unreliable but cheap, she may decide to perform it several times in a row to increase her confidence in the outcome. Third, to disambiguate the negative feedback obtained after a contingent action does not lead to the expected outcome, the decision maker must consult a causal model of the problem under consideration [8, 9]. For example, the engineer must use her knowledge about how the change of a component will impact the plant’s output to select a subsequent test or remedial action.

Decisions that require disambiguating a latent state through a series of information-seeking actions belong to the broad class of partially-observable Markov decision processes (POMDPs) [4, 5]. For problems with fully observable states, like video games [10, 11] or route planning [12], finding optimal actions boils down to a search over action sequences, in which the optimal action *a*_*i*_ at time step *i* only depends on the agent’s current state (e.g., the location in a maze). While the search is often intractable because the number of possible paths grows exponentially with the number of state variables and the planning horizon, advances in artificial intelligence have identified clever heuristics that allow automated solvers to derive action policies directly from compact descriptions of the problem and scale up to problems involving many millions of states (see [13] for a review). In contrast, in POMDPs the decision policies take the form of a sequence of actions and observations (*a*_1_, *o*_1_, *a*_2_, *o*_2_, …), and the next best action *a*_*i*_ may depend on the entire sequence, making these problems much more difficult to solve optimally [14]. For small problems the usual approach is to transform the POMDP over latent states into a fully-observable Markov decision process (MDP) over belief states–a probability distribution over the latent states–and solve it using Bellman’s equation [15, 16]. However, this solution works only for problems with few states and actions and a short planning horizon; for more complex problems, efficient planning must rely on heuristic strategies and relaxations that are less well characterized than their fully-observable counterparts.

Here we study how people decide which decisions to address to disambiguate a latent state and replan when an initial plan fail. We build on studies of simple perceptual decisions. A well-studied example is a decision about the net direction of motion of randomly moving dots [17, 18]. In such binary decisions (e.g., left-right motion), humans and monkeys accumulate noisy samples of evidence over time. This basic paradigm has been extended to study decisions that are structured hierarchically. Lorteije et al. [19] trained monkeys to solve an explicit decision tree with stochastic evidence at every branching point. They found that the first level decisions were biased towards the second level decision that was easier, which shows that the different sub-decisions were not made independently but rather influenced each other to maximize the expected reward. Recent studies have shown that a graded expectation of potential outcome (also known as confidence) plays a key role in action selection and credit assignment in decision hierarchies. When two sequential decisions must be resolved correctly to receive a reward, confidence in the accuracy of the first decision influences the speed-accuracy tradeoff for the subsequent decision to maximize reward rate [20]. Confidence also helps disambiguate the cause of errors when they may be due to misperception or a covert change in the stimulus-response contingency [21, 22]. These studies are limited to the case of a single stream of evidence and used tasks in which the decision makers had no control over the evidence that was presented to them, and therefore these tasks do not require planning over sequences of actions.

We studied a novel task in which participants made a series of binary perceptual decisions arranged in a decision tree with stochastic evidence at each bifurcation. Decisions varied in difficulty, and people had to explore the decision tree until finding a target hidden at one of the lowest-level nodes of the decision tree (termed leaf nodes). Negative feedback obtained after choosing an incorrect leaf node provided ambiguous evidence about the cause of the error. Given the complexity of the problem (with 10^7^ possible states), the task cannot be solved by off-the-shelf POMDP solvers, but people were able to perform with high levels of accuracy. They did so by adopting a planning strategy based on a small set of heuristics. These include discarding information deemed unreliable to make a decision; a bias towards resolving uncertainty locally, collapsing probabilistic information into a categorical decision rather than carrying forward entire probability distributions; and the use of confidence to disambiguate negative feedback after an error. These results extends the framework of perceptual decision making to more complex decisions that comprise a hierarchy of sub-decisions.

## Results

### Hierarchical decision making task

**Fig 1:**
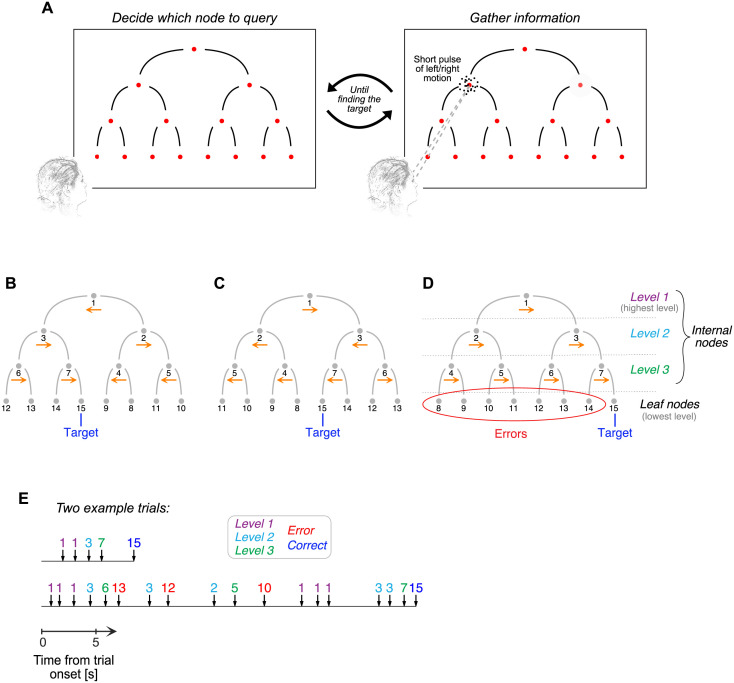
Hierarchical decision-making task. (A) Stimulus display. Participants had to identify which of the 8 lower-level nodes delivered a positive reward and ended the trial. Directing the gaze to a node from the top three levels of the decision tree and pressing a key triggered a short pulse of random-dot motion. The direction (left or right) and strength of motion assigned to each internal node were randomly selected in each trial. The target could be identified by following the correct direction of motion at each bifurcation from the root node to a leaf node. Participants were free to explore the decision tree as they wished to maximize the number of points earned. (B–C) Correct direction of motion at each internal node for two example trials. The true direction of motion is indicated by the horizontal arrows shown below each internal node. The numbers assigned to the nodes do not represent spatial positions but depend on the correct direction of motion at each bifurcation (as explained in the next panel). (D) Adopted nomenclature. Levels 1 to 3 are internal nodes. The leaf nodes are those at the lowest level of the decision tree. Nodes are numbered depending on the correct direction of motion at each bifurcation. If rightwards were the correct direction of motion at every bifurcation (as indicated by the horizontal arrows), the number assigned to each node would increase from top to bottom and from left to right, as indicated in the panel. (E) Example of sequence of choices from two representative trials. The vertical arrows indicate when a node of the decision tree was queried. The numbers above the arrows identify the node that was queried, following the convention described in panel D.

Four human participants were presented with a binary decision tree that bifurcated three times. They were tasked with finding a target hidden at one of the leaf nodes of the decision tree (Fig 1A). Each internal node of the decision tree was assigned a direction of motion, which could be rightward or leftward. The target was hidden at the leaf node identified by tracing the path defined by following the correct direction of motion at each bifurcation, starting from the root node of the decision tree. Fig 1B and 1C illustrates two example configurations, where the arrows (not shown to the participants) indicate the correct direction of motion at each bifurcation.

The participants were not informed about the correct direction of motion at each bifurcation, but they could infer it by querying the internal nodes of the decision tree. When participants directed their gaze to one of the internal nodes followed by a button press, they were presented with a short pulse (227 ms) of random dot motion (Fig 1A). The net direction of motion of the dots was either rightward or leftward, and coincided with the true direction of motion assigned to that node.

The difficulty of motion discrimination could vary from node to node. As usual in experiments using the random-dot motion stimulus, difficulty was controlled by the probability that each dot is displaced in the direction of motion as opposed to randomly. We refer to this probability as motion strength. The motion strength and direction (left/right) were sampled independently for each internal node of the decision tree, and were fixed for the whole trial.

Choosing a leaf node did not provide any motion information. Instead, if the chosen leaf node was the target, the trial ended and participants received a positive reward. If the chosen leaf node was not the target, the participant received negative feedback (a low-pitched sound) and had to continue exploring the decision tree until finding the target. A screen recording of the experiment is shown in a companion video (S1 Movie).

Participants were free to decide how to explore the decision tree to maximize the number of points earned and were informed in advance about the reward contingencies. Participants lost 1 point every time they queried an internal node for motion information, lost 3 points every time they chose a leaf node and it was not the target, and earned 10 points when finding the target. To encourage the search for strategies that lead to high rewards, participants received feedback about how many points they obtained after each trial. Also, at the end of each block of 50 trials, participants were informed about the total number of points obtained in the block and in all previous blocks.

Throughout the manuscript, we use numbers to identify each node of the decision tree. The numbers do not reflect spatial positions, but rather depend on the true direction of motion at each bifurcation (Fig 1D). Nodes 1 through 7 are internal nodes and nodes 8 through 15 are leaf nodes. Node 15 is the target, and nodes 1, 3, and 7 are the nodes at levels 1–3 that are on the path to the target. A simple way of thinking about the numbering convention is that if the true direction of motion were rightward at every internal node, then the numbers assigned to each node would increase from top to bottom and from left to right (Fig 1D).

Fig 1E shows two trials that exemplify the sequence of choices within a trial. The trial at the top illustrates a typical one, in which the participant made no errors before finding the target but queried the root node twice. Fig 1E (bottom) shows a trial in which the participant made multiple errors before finding the target, both at internal and leaf nodes. The four participants made an average of 6.5, 7.7, 9.4, and 7.7 queries per trial, respectively (all standard errors smaller than 0.2).

### Motion choices depend on motion strength and tree level

**Fig 2:**
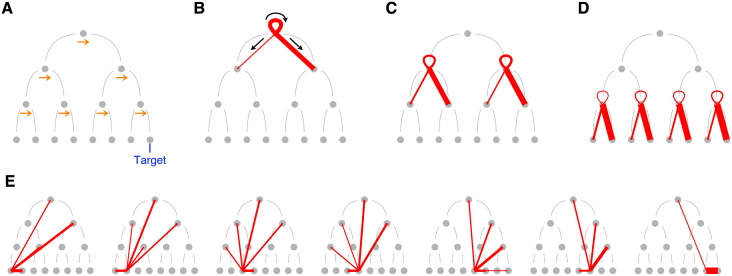
Transition probabilities between nodes of the decision tree. (A) In all the panels, the nodes of the decision tree were re-arranged following the convention depicted in Fig 1D. The re-arrangement can be interpreted as-if rightward were the true direction of motion at every bifurcation (indicated here by the right-pointing arrows). (B-E) Red lines identify the most frequent transitions between pairs of nodes. Transitions from nodes at levels 1–3 are shown in panel B–D, and transitions from leaf nodes are shown in panel E. The width of the line from node *x* to node *y* is proportional to the probability of transitioning from *x* to *y* given that last query was to node *x*. Unlikely transitions (conditional probability < 0.075) were omitted. (B–D) After querying an internal node, the more frequent actions were choosing one of the two child-nodes, or re-querying the same node. The ribbon corresponds to re-queries. Because of the notation convention, the true direction of motion is rightward at every internal node. (E) From left to right, transitions from leaf nodes 8 to 14. We excluded the target (node 15) since querying it terminates the trial.

Before analyzing how multiple decisions are chained into a sequence, we focus on the choices made after querying the internal nodes of the decision tree. Almost all trials (>99.7%) started with a query at the root node. The three most common actions after querying an internal node were to query the same node again (which we refer to as a re-query), or to query one of the two child-nodes (Fig 2A–2D). These actions represent the vast majority of the actions that follow the query of an internal node (99%, 97%, 98% and 98% for participants 1–4, respectively). The action that immediately follows an error at a leaf node was less stereotypical and could be directed to different nodes of the decision tree, including other leaf or internal nodes (Fig 2E). In this section we focus on the subset of queries for which after observing the random-dot motion stimulus, participants either chose one of the two child nodes or requested additional information from the same node; as just mentioned, these are the most frequent actions after querying an internal node.

**Fig 3:**
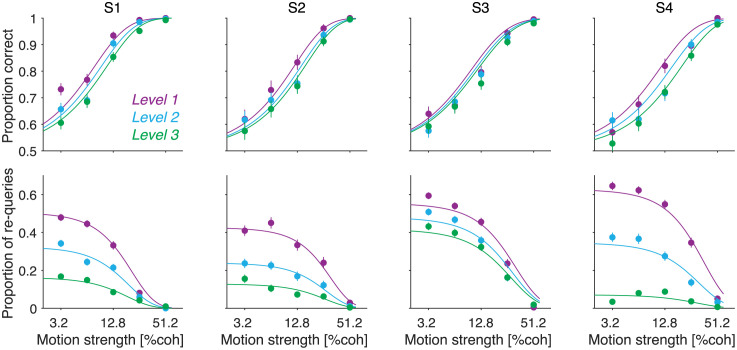
Motion choices depend on motion strength and tree level. The top row shows the proportion of correct motion choices as a function of motion strength. The bottom row shows the proportion of queries that were followed by another query at the same node. Solid curves are fits of a detection model. Decisions made at different levels of the decision tree are displayed in different colors. Each column shows data from one participant (S1 to S4). Error bars indicate s.e.m.

The accuracy of the decisions made at internal nodes depends on the node’s motion strength and its level in the decision tree. Accuracy was calculated from those queries that were directly followed by the selection of one of the two child nodes: a motion choice is considered correct if the chosen child node is in the true direction of motion. Unsurprisingly, stronger motion led to more accurate motion choices (Fig 3, top row) (Eq 6, *β*_1_ = 15 ± 0.6, *p* < 10^−8^). More interesting is the lawful relationship between the accuracy of motion choices and tree level. Motion choices were most accurate at the highest level of the decision tree, and were least accurate at the lowest level of the decision tree (Fig 3, top row) (Eq 6, *β*_2_ = −2 ± 0.26, *p* < 10^−8^). The relationship between accuracy and tree level cannot be explained by the properties of the motion stimuli, which were statistically identical across tree levels.

After querying an internal node, participants often queried the same node again (termed a re-query). Just as the accuracy of the motion choices, the frequency of re-queries depended on the strength of motion and tree level. Re-queries were more likely when the motion was weak (Fig 3, bottom row) (Eq 7, *β*_1_ = −7 ± 0.17, *p* < 10^−8^). The frequency of re-queries also depended on the level of the decision in the decision tree: participants were less likely to query the same node again for decisions deeper in the decision tree (Fig 3, bottom row) (Eq 7, *β*_2_ = −0.7 ± 0.02, *p* < 10^−8^).

### A detection model explains the decisions made at internal nodes

The solid lines in Fig 3 are fits of a detection model inspired by probability summation [23] and early models of decision making [24]. A key aspect of these models is that a sample of evidence from an evidence stream is compared to a criterion to decide if the evidence sample is reliable enough to make a detection [23] or discrimination [24] judgment. If the evidence exceeds a criterion, a choice is made; otherwise, the evidence sample is discarded and a new sample is obtained from the evidence stream. We extended this class of models to decisions involving active sampling of information from a decision hierarchy, and compared the model to one in which evidence samples were integrated across successive queries as prescribed by optimal models of decision making [25]. To foreshadow our results, they favor the model without integration, which we refer to as the detection model [26].

**Fig 4:**
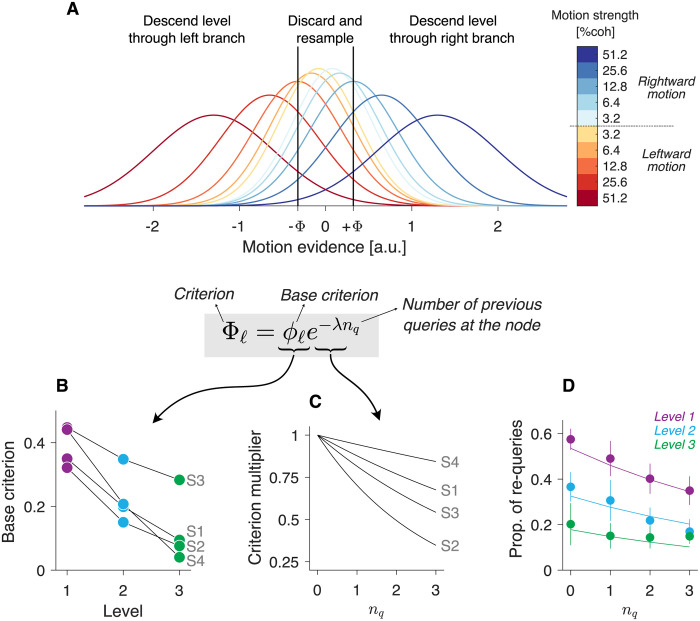
A simple detection model explains the motion choices. (A) Distributions from which the momentary motion evidence were sampled. The momentary motion evidence is normally distributed, with mean and variance that scale linearly with motion strength. The sign of the mean depends on motion direction. The slopes were fit independently for each participant (here we show the distributions from participant 1). The decision is made comparing an evidence sample against two criteria located at ±Φ. The criterion is given by the product of a base criterion, *ϕ_ℓ_*, and a term that decays exponentially with the number of successive queries made at the node (*n*_*q*_). (B) Base criterion for every participant and level of the decision tree, obtained from the best-fitting model. (C) The criterion, Φ, approaches zero exponentially as the number of successive queries at a node (*n*_*q*_) increases. Decay rate is determined by λ. Each curve depicts the best-fitting exponential function for each participant. (D) Frequency of re-queries, as a function of the number of previous successive queries at the node (*n*_*q*_). This proportion decreases with *n*_*q*_ and with the level of the decision in the tree (indicated by the different colors). Solid lines are predictions from the detection model. Only the lower motion strengths (below 25.6%) were included in this analysis. Error bars indicate s.e.m. across participants.

We modelled the representation of the momentary motion evidence with a gaussian probability density function. The mean and the variance of the momentary evidence is a linear function of motion strength (Fig 4A). The sign of the mean depends on the true direction of motion: positive for rightward motion, and negative for leftward motion. Two decision criteria distributed symmetrically around zero (vertical lines in Fig 4A) divide the domain of the momentary evidence in three regions. If the evidence falls below the leftmost criterion, the decision maker interprets that the net direction of motion was leftward and descends a level through the left branch of the decision tree. Likewise, if the evidence falls above the rightmost criterion, the decision maker descend levels through the right branch. If the evidence falls between the two criteria, the evidence sample is discarded, and the same node is queried again.

In our model, the decision criterion depends on (i) the level of the decision in the decision tree, and (ii) the number of successive queries at the node. We model this dependency as the product of a base criterion that depends on the level *ℓ* of the decision tree, *ϕ*_*ℓ*_, and a term that decays exponentially with the number of re-queries (Fig 4A, bottom). The model has 6 parameters that were fit by maximum likelihood to the choices made after querying the internal nodes of the decision tree.

The predictions of the best-fitting model are shown by the solid lines in Fig 3. The model provides a very good fit to the behavioral data, capturing the influence of motion strength and tree level on the proportion of correct motion choices and re-queries. In the best-fitting model, the base criterion decreased with tree level for every participant (Fig 4B), implying that stronger motion was needed at the higher tree levels to commit to a motion choice. This explains why more re-queries were made at the higher levels of the decision tree (Fig 3, bottom row) and why the motion choices made at the higher levels were on average more accurate than those made at the lower levels of the decision tree (Fig 3, top row).

The criterion also depended on the number of re-queries. It becomes gradually closer to zero as more queries were made at a node (Fig 4C). This explains why the probability of making a re-query decreased with the number of past re-queries (Fig 4D). The effect is analogous to the collapse of the decision bounds in reaction-time experiments, which is optimal when the reliability of the evidence is unknown to decision makers [27].

### No integration of motion information across re-queries

**Fig 5:**
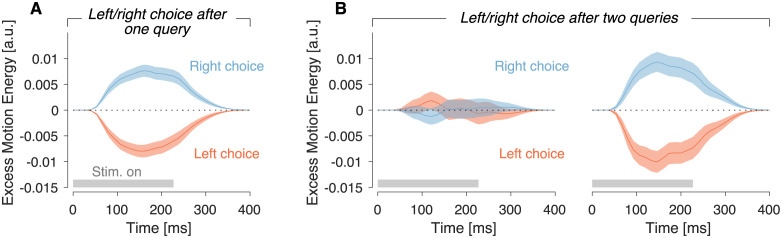
Only motion information from the last query informs the left/right choice. (A) Influence of motion energy residuals on the decision to descend levels through the left or right branch. The residuals were calculated by applying a filter to the sequence of random dots and subtracting the mean of all stimuli of the same motion strength and direction. Positive (negative) residuals indicate an excess of motion in rightward (leftward) direction. For left/right choices made after a single query, the motion energy residuals were positive for rightward choices and negative for leftward choices. (B) As panel A, except that we analyze the left/right choices made after two successive queries of the same internal node. The two panels show the motion energy residuals obtained from the first and second queries, sorted by the left/right choice made after the second one. Only the motion energy residuals from the second presentation distinguished between leftward and rightward choices, which indicates that the motion information from the first query did not influence the ultimate choice. Shading indicates s.e.m. The latency introduced by the impulse response of the motion energy filters explain the offset between the time of stimulus presentation (gray horizontal bars) and the onset of motion selectivity (Methods).

A strong assumption of the detection model is that weak motion evidence is discarded, unlike optimal models of decision making which posit that all evidence bearing on a decision should be integrated [25]. An analysis of the stimulus information used to make the decision—what is known as psychophysical reverse correlation or kernel analysis [28, 29]—supports this hypothesis. Fig 5 displays the degree to which the variability in the noisy display affects the left/right choice made after querying an internal node. For motion choices made after just one query, the psychophysical kernel was significantly positive (negative) for rightward (leftward) choices, indicating that the choice was guided by the motion information in the stimulus (Fig 5A)(*p* < 10^−8^, likelihood-ratio test, *H*_0_: *β*_3_ = 0, Eq 9). For motion choices made after two successive queries, the psychophysical kernel calculated using the motion information from the second query was also significantly different from zero (Fig 5B, right)(*p* < 10^−8^, likelihood-ratio test, *H*_0_: *β*_4_ = 0, Eq 10). However, the motion information from the first of the two queries had no influence on the eventual left/right choice (Fig 5B, left; *p* = 0.54, *H*_0_: *β*_3_ = 0, Eq 10) and a comparison of nested regression models favored the model without motion information from the first motion pulse (Eq 10, Δ*BIC* = 7.4 supporting the model without the *β*_3_ term). A similar conclusion was reached by analyzing the motion energy kernels separately for the 3 levels of the decision tree (S1 Fig). Had motion information been accumulated over the two motion pulses, both pulses should have been informative about the ultimate choice [30].

To confirm the results of the model-free analysis of the motion energy, we fit a model identical to the detection model except that the motion evidence was accumulated over successive queries. For example, if the evidence obtained after a query, *e*_1_, falls between the two criteria at ±Φ_*ℓ*_, the node is queried again and the decision is then based on *e*_1_ + *e*_2_, where *e*_2_ is the evidence obtained from the second query. If *e*_1_ + *e*_2_ again falls between the two criteria, another query is made (*e*_3_) and then *e*_1_ + *e*_2_ + *e*_3_ is compared against the criteria. The integration model has the same number of parameters as the detection model. A comparison of the best-fitting models favored the detection model over the integration model, confirming the results of the model-free analysis (∑ Δ*BIC* = 43 across participants; S2 Fig).

We used model simulations to confirm that if participants were accumulating evidence across successive pulses of motion, we would have observed an influence of a motion pulse on choice even if it was followed by a re-query. To this end, we simulated the detection and integration models that best fit the data. As we did for the data, we select the subset of queries for which a left/right choice was made after two successive queries, and repeat the regression analysis reported in the previous section. For simulations of the integration model, both pulses had a significantly positive influence on the ultimate choice (both *p*′*s* < 10^−4^, Eq 10; Δ*BIC* = 5.5 in favor of the full model). For the detection model only the last one was informative about the ultimate choice (*p*_1*st*_ = 0.71 and *p*_2*nd*_ < 10^−8^, Eq 10; Δ*BIC* = 5.5 in favor of the model without the *β*_3_ term). Together with the motion-free analysis of the motion energy and the formal model comparison, the analysis supports the conclusion that participants did not integrate motion information across re-queries.

We compared the detection model against other alternative models. A common assumption in models of behavior in the random-dot motion task is that the variance of the momentary motion evidence is independent of motion strength (e.g., [31]). We compared our detection model against one in which the variance does not depend on motion strength. For two of the four participants, a model comparison favored the detection model in which variance scales with motion strength; for the other two participants, the difference in BICs was too small to favor any one model (S2 Fig). Using the Akaike Information Criterion instead of the BIC led to the same conclusion. These data are consistent with previous studies that found that scaling the variance of the noise with motion strength provides a slightly better fit to choice [32] and response-time [33].

We also evaluated the possibility that tree level affects other parameters of the model besides the criteria Φ_*ℓ*_. For instance, if participants paid more or less attention to a decision depending on tree level, the signal-to-noise might differ across levels and contribute to the differences in performance that we observed (Fig 3). We tested two alternative models in which the signal-to-noise parameter (*κ*) was allowed to change across tree levels. In one, *κ* depended on the level in the decision tree, but the criterion Φ was the same across tree levels (model ‘1Φ3*κ*’ in S2 Fig). In another variant, both *κ* and Φ depended on tree level (model ‘3Φ3*κ*’, S2 Fig). A model comparison favored the detection model over these two alternatives (S2 Fig).

Because participants start each trial exploring the decision tree from top to bottom, there is a correlation between tree level and the order of a query in the trial. Therefore, it is possible that query order (and not the level of the decision in the decision tree) influences the placement of the criterion, Φ, that controls the probability of a re-query. To evaluate this possibility, we fit an alternative model in which the placement of the criterion depends on the order of the query in the trial. In the model, the criterion decreases exponentially from Φ_0_ to *ϕ*_∞_. The decay rate, Φ_0_ and *ϕ*_∞_ are free parameters of the model. A BIC analysis strongly favored the detection model in which the criterion explicitly depends on tree level (S2 Fig). This conclusion was also supported by a model-free analysis, which shows that the relation between tree level and the probability of making a re-query is not mediated by query order or time elapsed in the trial (S3 Fig). Taken together, the comparison against different alternative models supports the hypothesis that the differences in performance across levels of the decision tree is explained by the influence of tree level on the placement of the criterion that determines if the motion evidence is reliable enough to commit to a motion choice.

### Failure of a probabilistic model to account for human behavior

We aimed to compare the participants’ behavior to that expected under the optimal policy. However, finding the optimal policy in our task is computationally challenging. Since each node can be in 10 states (5 motion strengths × 2 directions of motion) and there are 7 internal nodes, the number of possible problem states is 10^7^. Because the true state is not fully observable, optimal decision-makers must represent a probability distribution over the problem states, and update it with the motion information they gather and the errors they make as they explore the decision tree. To find the action that maximizes the expectation of future rewards, decision-makers should plan over a potentially infinite sequence of possible future actions and observations. While for low-dimensional problems the optimal solution can be approximated by transforming the POMDP over the unobserved states into an fully-observable MDP over the belief states and solve it using Bellman equation, in our task the state space is too large for this approximation to work.

Because of these challenges, we relied on simulations to approximate the optimal decision policy. The Bayesian model represents a probability distribution over the problem states, P(s|E,V), where *s* is defined by the motion strength (*c*) and motion direction (*d*) at each of the 7 internal nodes, *s* = (*c*_1_, *d*_1_, *c*_2_, *d*_2_, …, *c*_7_, *d*_7_). The tuple *E* = (*E*_1_, *E*_2_, …, *E*_7_) contains all the motion samples obtained from past queries at the 7 internal nodes; *E*_*i*_ is the set of motion samples obtained from queries at internal node *i*. *V* is the set of leaf nodes that were already queried and turned out not to be the target.

Before an error at the leaf nodes (i.e., when *V* is the empty set), P(s|E,V) can be factorized as,

$$\begin{array}{c} P\left(s|E,V\right)=\prod_{i=1}^{7}P_{i}\left(c_{i},d_{i}|E_{i}\right)\;, \end{array}$$ (1)

where $P_{i}\left(c_{i},d_{i}|E_{i}\right)$ is the probability that internal node *i* has motion strength *c*_*i*_ and direction *d*_*i*_, given the set of motion samples *E*_*i*_ obtained from previous queries at node *i*. The values of *c*_*i*_ and *d*_*i*_ are those that correspond to state *s*.

This factorization is no longer valid after an error at a leaf node (i.e., when *V* is no longer empty). Leaf nodes are what in the language of Bayesian networks are called colliders, and conditioning on one renders a statistical dependency between its otherwise independent parents [9]. Because of this, the motion information obtained from one internal node should affect the decision-maker’s beliefs about the direction of motion at other nodes of the decision tree. We can illustrate this with an example, for the stimulus configuration shown in Fig 1B. Imagine that the decision-maker is completely certain about the motion choices made at nodes 1 and 3. If the decision-maker queries node 14 and receives negative feedback, then the decision maker can be almost certain that the true direction of motion at node 7 is rightward, even if she has not yet queried that node; the alternative is that the motion choices made at nodes 1 and 3 were wrong, but these choices were made with high certainty. The dependency between the motion direction at the different internal nodes introduced by the colliders renders the update of P(s) with motion information less straightforward. In Methods we explain how we factorize and simplify the update of P(s|E,V) when *V* is not empty; here we focus on the planning problem of how the Bayesian model selects the next best action.

Before the selection of every action, the agent uses an internal model of the task to estimate how costly it will be to find the target starting from each of the 15 possible next actions. The agent samples a state, s*, from the posterior distribution over states P(s|E,V). It then assumes that s* is the true state of the system. The state s* identifies the target, T*. The agent then simulates random actions, starting with action *a*, until reaching T*, with the following constraint. If a query is to be made at a leaf node, the leaf node to be queried is the one with the highest probability of being the target. A copy of the posterior distribution over states P(s) is updated after each imaginary observation, which the agent uses to determine which leaf nodes has the highest probability of being the target. The agent repeats this process 2, 000 times for each of the 15 possible next actions, *a*. For each simulation, the agent computes the cost incurred in finding the target, and the action to execute next is the one for which the expected cost is minimized.

All the process occurs ‘in the head’ of the decision maker, only once the next best action is identified is the action executed ‘in the world’. Then the distribution P(s|E,V) is updated with the observation obtained from the environment, and the whole simulation-based procedure is repeated to determine the next best action.

**Fig 6:**
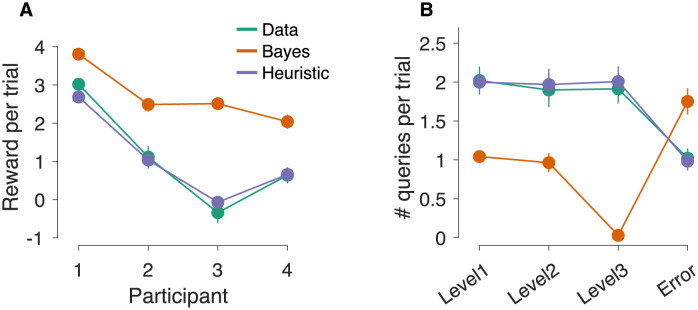
Comparison of average reward and number of queries between data and models. (A) Average reward per trial obtained by the participants, the Bayesian model and the heuristic model. Error bars indicate s.e.m. across trials. (B) Average number of queries per level and of errors at leaf nodes. The averages were first calculated per participant and then across participants. Error bars indicate s.e.m. across participants. The predictions of the Bayesian model (Heuristic model) are based on 2,000 (50,000) simulated trials per participants.

In summary, we developed a method to approximate the optimal decision policy for our task. While we cannot guarantee that the approach described above (and in more detail in Methods) allowed us to find the optimal policy, the decision policies derived with it lead to significantly higher rewards than those obtained by the participants (Fig 6A). Because the Bayesian model uses the signal-noise parameters from the detection model fit to the data, the difference in reward income cannot be explained by differences in sensitivity to motion information but must be the result of differences in strategy.

**Fig 7:**
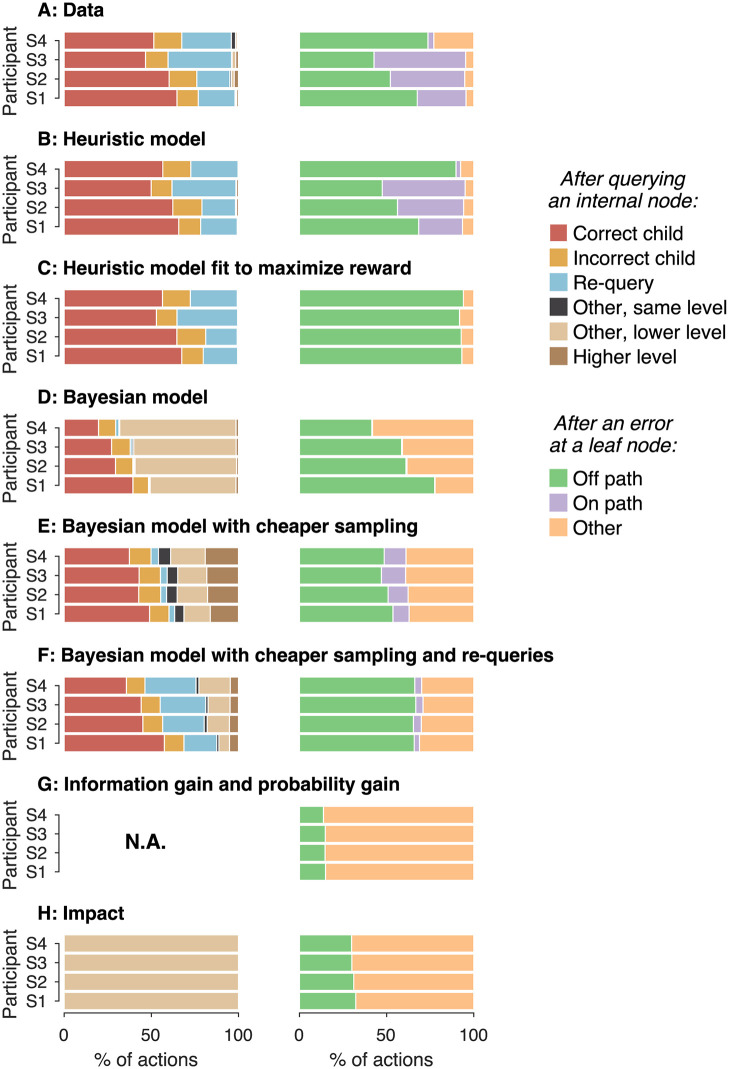
Distribution of actions after the query of internal and leaf nodes. The left column shows the types of action selected after a query of an internal node, and the right column shows those selected after an error at a leaf node. Panels A–H represent different datasets, identified in each panel. We sorted the actions that follow the query of an internal node into 3 categories: *on-path* queries, *off-path* queries, and queries that cannot be classified as neither on- nor *off-path* (‘other’). We sorted the actions that follow an error at a leaf node into 6 categories: choosing the child node that is in the true direction of motion (‘correct child’), the other child node (‘incorrect child’), querying the same node again (‘re-query’), other nodes at the same level excluding re-queries (‘other, same level’), nodes of lower level that are not direct child nodes (‘other, lower level’), and nodes located at higher levels of the decision tree (‘higher level’).

We observed many qualitative differences between the behavior of the Bayesian model and the data that explain the difference in reward. The Bayesian model made fewer queries at internal nodes and more errors at leaf nodes than the participants (Fig 6B). That is, the balance between information-seeking and reward-seeking actions was different for model and data. Further, the Bayesian model almost never queries the same internal node more than once in a row (Fig 7D, left), unlike the participants who made many re-queries (Fig 7A, left).

**Fig 8:**
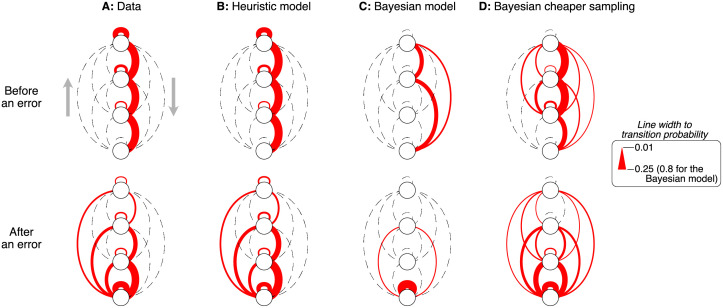
Participants and heuristic model made similar transitions between levels. Transition frequencies between levels of the decision tree, obtained by grouping all nodes from the same level. The top row shows the transitions before making an error at a leaf node. The bottom row shows the transition after at least one error at a leaf node. The width of the red lines is proportional to the transition frequency. The dashed lines are placeholders for infrequent transitions. The last column corresponds to a Bayesian model in which the cost of querying internal nodes was reduced to 30% of its true value. In each of the 8 panels, transitions to a lower level are shown on the right, and transitions to a higher level are shown on the left. The ribbon identifies transitions between nodes at the same level of the decision tree (including re-queries). Transition probabilities were first calculated per participant, and then averaged across participant (see Methods).

Additional differences between model and data can be seen in Fig 8, which shows the transition probabilities between levels, obtained by grouping nodes within each level before calculating the transition probabilities. The width of the red lines is proportional to the transition probability. Transitions from higher to lower levels are shown on the right of each graph, and transitions in the opposite direction are shown on the left. In the data, the action that follows an error can target different levels of the decision tree. After querying an internal node, transitions are often to the same level or to the level immediately below. This is observed both before and after an error at a leaf node. In contrast, the Bayesian model skips nodes from tree level 3, going straight from level 1 or 2 to the leaf nodes. After an error at a leaf node, the Bayesian model usually transitions to other leaf node and rarely goes back to querying an internal node (Fig 8).

It may be surprising that the Bayesian model does not query level 3 nodes. We can provide some intuition as to why this is optimal. Imagine that the probability of making a correct motion choice is 0.8 after querying an internal node, and 0.5 (chance) if the node is not queried. And let’s assume for simplicity that motion choices at levels 1 and 2 were made with full confidence. Should the decision maker query a level 3 node before selecting a leaf node? If queried, the expected cost is (1–0.8) × (-3) = -0.6, since there is a probability of (1–0.8) of picking the wrong leaf node and lose 3 points before choosing the target. On the contrary, if the level 3 node is not queried, the expected cost is 0.5 * (-3) = -1.5, because on average half the time the decision maker would choose the wrong leaf node and lose 3 points. The difference between the two expected costs is less than the cost of making a query at the internal node (1 point), so the best strategy in this case is not to query the node from level 3 of the decision tree. By a similar reasoning it can be shown that it is convenient to make queries at the nodes of level 1 and 2, since these allow the decision maker (in the best of cases) to discard more than one leaf node. Of course, the convenience of querying level 3 nodes depends on the payoff structure and the sensitivity to motion information; later we will see a case in which the cost of querying the internal nodes is reduced and the decision policy derived with the Bayesian model includes the query of level 3 nodes.

A possible explanation for why the Bayesian model made fewer queries at internal nodes and more errors at leaf nodes than the participants, is that subjective rewards were different from the true ones. This would be the case if, for instance resolving uncertainty about motion direction were considered rewarding regardless of the task goals (e.g., [34]). We reasoned that the Bayesian model could behave more similarly to data if the cost of querying internal nodes were reduced. To test this hypothesis, we applied the Bayesian model to a case in which the cost of querying an internal node was only 30% of the value we used in the experiment. In this case, queries at internal nodes were more evenly distributed between levels, more similarly to what was observed in the data (S4(A) Fig). However, the Bayesian model still behaved very differently from the participants. The Bayesian model performed very few successive queries on the same internal node, frequently transitioned from lower-level to higher-level internal nodes, and made lateral transitions between different nodes from the same tree level (Fig 7E and S4(B)–S4(E) Fig). The differences between the data and the Bayesian model are observed both before and after an error at a leaf node, which indicates that the differences between them are not just due to differences in how blame is assigned after an error (Fig 8A and 8D).

A salient aspect of the data is that the participants made more re-queries at the higher levels of the decision tree than at the lower levels (Fig 3, bottom row). This seems to be a sensible strategy since misjudgments made at the higher tree levels can cause the decision maker to spend a long time exploring the wrong branch of the decision tree. To determine if this is indeed a sensible strategy, we solved the Bayesian model for a case in which the sampling cost was reduced to 50% of its true value for queries of internal nodes and to 5% for re-queries. The goal of this manipulation was to increase the number of re-queries made by the Bayesian model to determine if it is better to make more re-queries at the higher levels or the lower levels of the decision tree. As expected, changing the reward structure in this way led to more re-queries, in a number comparable to those observed experimentally (Fig 7F). When analyzing the number of re-queries by tree level and motion strength, we observed that the Bayesian model performed more re-queries at the lower levels than at the higher levels of the decision tree (S5 Fig). This is opposite to what we observed in the data. Therefore, if the large number of re-queries that we observed in the data were due to their subjective cost being lower than the query of other nodes, participants should have made more re-queries at lower levels of the decision tree than at higher levels.

Taken together, these results show that the behavior derived from the Bayesian model is qualitatively different from that shown by our participants. This failure motivated the development of the heuristic model that we present below.

### Shallow sampling norms

Given that the performance of the Bayesian model was superior to that of the participants, we studied if simpler metrics could better capture the participants’ behavior. We adopted metrics frequently used in the active sensing literature to arbitrate between information-seeking actions (see [35] for an overview). These metrics are myopic, shallow or greedy, in that they only look one step into the future. They do not take into account information about the costs and rewards of the different actions and outcomes; instead, actions are prioritized based on the expected change in the posterior probability distribution over task-relevant categories. The best next action is the one that maximizes a scoring function that is different depending on which metric is used. We explore three well-studied metrics: probability-gain, information-gain and impact.

These metrics were applied to the probability distribution B(T|E,V), which is the belief that leaf node *T* is the target given all past motion observations *E* = {*E*_1_, *E*_2_, …, *E*_7_} obtained from the 7 internal nodes and the set *V* of leaf nodes that were already visited and turned out not to be the target.

For probability-gain (PG) the score associated with each possible action is given by the expected change in the peak of the posterior:

$$\begin{array}{c} {\text{score}}_{\text{PG}}\left(a|E,V\right)={\left\langle \underset{T}{\text{max}}(B(T|a,o,E,V))\right\rangle }_{P(o|a,E,V)}-\underset{T}{\text{max}}(B(T|E,V)), \end{array}$$ (2)

where actions *a* are the 15 available actions and max_*x*_ is the maximum over the 8 leaf nodes. The expectation is calculated over the possible observations *o* that follow action *a*. If *a* is the query of an internal node, the observations *o* are motion samples. If *a* is the query of a leaf node, the observations *o* can take only two values depending on whether the leaf node is, or is not, the target.

The scoring function for information-gain (IG) is the expected reduction in the entropy of B(T|E,V) following action *a*:

$$\begin{array}{c} {\text{score}}_{\text{IG}}\left(a|E,V\right)=H[B(T|E,V)]-{\left\langle H[B(T|a,o,E,V)]\right\rangle }_{P(o|a,E,V)}, \end{array}$$ (3)

where *H* denotes entropy (a measure of uncertainty [36]).

The scoring function for the impact metric (I) is given by the expectation of the sum absolute change in B(T|E,V) after action *a*:

$$\begin{array}{c} {\text{score}}_{\text{I}}\left(a|E,V\right)=\sum_{T}{\left\langle \left|B\left(T|a,o,E,V\right)-B\left(T|E,V\right)\right|\right\rangle }_{P(o|a,E,V)}, \end{array}$$ (4)

where || denotes absolute value and the summation is over the 8 leaf nodes.

None of these metrics led to behavior similar to that of the participants. The decision policies derived from probability-gain and information-gain ignored the internal nodes and searched directly over the leaf nodes (Fig 7G). This strategy leads to low reward in our task (-0.5 points per trial on average). Policies derived with the impact metric did much better, leading to an average reward per trial of 2.06 ± 0.14 across participants. In this case, the strategy consisted of making only one query at the root node, followed by randomly selecting leaf nodes from the branch favored by the first query (Fig 7H). These strategies are clearly different from that adopted by our participants.

Note that instead of applying the different sampling norms to the beliefs B(T|E,V), we could have applied them to the posterior probability over motion strength and direction at the internal nodes, $P(c_{1\ldots 7},d_{1\ldots 7}|E,V)$. Then the sampling norms would favor the exploration of nodes for which the uncertainty is high (i.e., the ones that have not been visited before). This could be a sensible strategy if participants were asked to explore the decision tree freely without any task instruction, but is clearly not the strategy followed by our participants and thus we did not explore this possibility any further. Taken together, our analyses shows that the shallow sampling norms commonly used in the active-sensing literature could not account for the participants’ behavior in our hierarchical decision-making task.

### Heuristic model

In previous sections we saw that neither a Bayesian model nor one based on commonly used sampling norms could explain the participants’ behavior in the task. In this section we present an alternative model which is based on a small set of heuristics of low computational complexity. The heuristic model extends the detection model with a mechanism to determine which node to query after an error, and could account for most aspects of the participants’ behavior.

The negative (auditory) feedback obtained after an error at leaf node ***T*** provides ambiguous evidence about the cause of the error. It informs the participant that an error was made in at least one of the nodes that connect the root node to ***T*** (termed the error path, ***P***_***T***_), but it does not indicate which of these three choices were wrong.

We hypothesize that decision makers would use the confidence in the motion choices to disambiguate the negative feedback obtained after an error at a leaf node. We define the confidence in having made a correct motion choice at internal node *i* as the probability that the choice is correct given the sample of motion evidence *e* obtained the last time that node *i* was queried. The confidence in that rightward is the correct direction of motion (i.e., *d* = 1) can be calculated using Bayes rule and marginalizing over motion strength *c*:

$$\begin{array}{c} {\text{conf}}_{i}^{+}=P_{i}\left(d=1|e\right)=\sum_{c}\frac{P\left(e|c,d=1\right)P_{i}\left(c,d=1\right)}{P(e)}\;. \end{array}$$ (5)

Confidence in a leftward choice is simply ${\text{conf}}_{i}^{-}=1-{\text{conf}}_{i}^{+}$. The likelihood P(e|c,d=1) is given by the probability density function of the normal distribution (Eq 14), and is plotted in Fig 4A for different motion strengths.

The heuristic model compares the confidence in the motion choices to decide which node to blame for an error and to choose a subsequent action. We define low-confidence choices as those for which confidence is below a criterion *ω*. If two decisions from the error path were made with low confidence, the model assigns the responsibility for the error to the highest level node among those belonging to ***P***_***T***_ whose motion choices were made with low confidence. For example, if an error was made at node 11, and the motion choices at nodes 2 and 5 were made with low confidence, then the model holds node 2 responsible for the error, since (i) node 2 belongs to the error path ***P***_***T***_, (ii) the motion choice was made with low confidence, and (iii) node 2 is at a higher level of the decision tree than node 5. Then, the model performs a query on the node that was blamed for the error (node 2 in this case). The rationale here is that if the decision maker cannot determine with certainty which motion choice was incorrect (since more than one motion choice was made with low confidence), it is reasonable to re-query one of the nodes of the error path to resolve this ambiguity.

We use the term *on-path* to refer to those queries for which after an error at leaf node ***T***, the decision maker selects a node from the error path ***P***_***T***_. For example, querying nodes 1, 2 or 5 after an error at node 11 would be considered *on-path* queries.

On the contrary, if only one (or none) of the motion choices along the error path were made with low confidence, the model assigns responsibility for the error to the node belonging to the error path ***P***_*T*_ for which the motion choice was made with the least confidence. For example, if an error was made at node 11 and only the decision at node 2 was made with low confidence, then the responsibility for the error is assigned to node 2. The next action is to query the child node of the one that was held responsible for the error and that is not in the error path ***P***_***T***_. In the example, the decision maker would make a query on node 4 since it is the child of node 2 that was not on the path that led to the error on node 11. The logic here is that if only one decision in the error-path was made with low confidence, then it is reasonable to assume that the error was due to the choice made at that node and continue exploring the decision tree through the ‘counterfactual’ path (i.e., the path that would have been taken had the motion choice at that node been different from the one that was actually made).

We use the term *off-path* to refer to those queries for which after an error at leaf node ***T***, the decision maker selects the child node of one of the nodes in ***P***_***T***_ that was not itself in the error path. For example, querying nodes 3, 4 or 10 after an error at node 11 would be considered *off-path* queries.

In summary, we have proposed a mechanism by which the confidence in the motion choices is used to resolve the ambiguous feedback received after an error at a leaf node and choose a subsequent action. An additional assumption incorporated in the heuristic model is that once a node has been blamed for an error, that node cannot be blamed again until new evidence is obtained from it. We adopted this ad-hoc modeling assumption because without it there were instances in which the model repeatedly blamed the same node for an error, which impaired performance. Because of this assumption, it may occur that no node from the error-path can be blamed for an error, in which case the model chooses at random and with equal probability any of the unvisited leaf nodes.

Once the model identifies which node to query after an error, it continues to follow the rules of the detection model—just as before the error. The heuristic model has only one free parameter (*ω*). It was fit to match the proportion of *on-path* queries between model and data (purple bars in Fig 7A and 7B, right column). All other parameter values were inherited from the fits of the detection model. By coupling the detection model with a mechanism to determine which node to query after an error, we obtain a model that can perform the task and make behavioral predictions that we can contrast with the experimental data.

**Fig 9:**
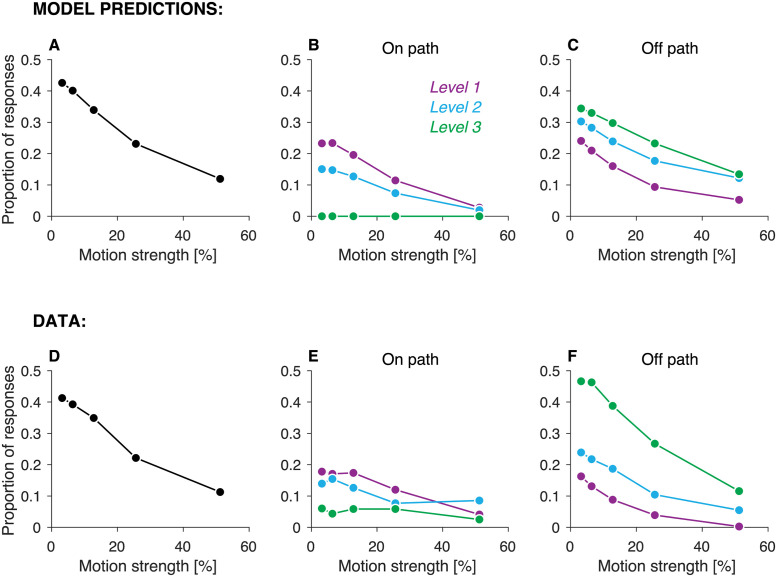
Motion strength and tree level influence which node is blamed for an error. Panels A-C show the predictions of the heuristic model and panels D-F correspond to the behavioral data. (A) Proportion of errors for which the blame was assigned to a node with the motion strength indicated in the abscissa, given that a node with that motion strength was in the error-path. Note that proportions do not need to add to 1 since the error-path can contain up to three different values of motion strength. (B) Proportion of errors that were followed by an *on-path* query to a node of level *ℓ*, given that level *ℓ* of the error-path had the motion strength indicated in the abscissa. For example, if the root node had the weakest possible motion strength, the probability that an *on-path* query is made at that node is ∼0.23. (C) Proportion of errors that were followed by an off-path query from a node of level *ℓ*, given that level *ℓ* of the error-path had the motion strength indicated in the abscissa. For example, if the root (level 1) node had the weakest possible motion strength, there is a ∼0.25 chance that the root node is blamed for the error and an off-path query is made to its child that was not on the error path. Proportions were first calculated per participant and then averaged across participants.

One prediction of the heuristic model is that the probability of assigning responsibility for an error to a node depends on the strength of motion of the nodes in the error path. This prediction is illustrated in Fig 9A. It shows the proportion of errors at leaf nodes in which the model blamed a node of motion strength *c* (shown in the abscissa), when a node with motion strength *c* was in the error path. The model was ∼4 times as likely to blame a node with the weakest motion than a node with the strongest motion. This ratio would be close to 1 if the blame were randomly assigned to any node in the error path. [Note that the proportions need not add to one since not all motion strengths are present on every trial, and the same motion strength could be present in more than one node.]

Another factor that ought to influence which node is blamed for an error is the level of a node in the decision tree. The predicted relationship between tree level and the probability that a node is blamed for the error differs for *on-path* and *off-path* queries. *On-path* queries are more frequently directed to the higher tree levels (Fig 9B). This is because at least two low-confidence decisions are required for the model to select an *on-path* query, and then the query is directed to the higher level node out of those for which the motion choice was made with low-confidence. In contrast, *off-path* queries are more frequently directed to the lower tree levels (Fig 9C). This is because decisions at the lower levels are made on average with weaker evidence than decisions at higher levels, since the criterion Φ_*ℓ*_ decreases with tree level (Fig 4B). As the blame for the error is assigned to the decision made with the least confidence, *off-path* queries usually target the lower levels of the decision tree.

These predictions were verified in the data. The blame for the error was more likely to be assigned to nodes with weak motion (Fig 9D)(*p* < 10^−8^, likelihood-ratio test, *H*_0_: *β*_1_ = 0, Eq 11). For *on-path* queries, the blame for the error was more likely to be assigned to the higher levels of the decision tree (Fig 9E)(*p* < 10^−8^, likelihood-ratio test, *H*_0_: *β*_2_ = 0, Eq 11). In contrast, for *off-path* queries the lower levels of the decision tree were more likely to be blamed for the error (Fig 9F)(*p* < 10^−8^, likelihood-ratio test, *H*_0_: *β*_2_ = 0, Eq 11).

As in the model, most actions that follow an error at a leaf node were either *off-* or *on-path* queries. While *off-path* and *on-path* queries represent only 6 of the 14 actions available after an error (excluding querying the same leaf node again), they represent most of the actions chosen by the participants after an error (96%, 95%, 95% and 77% for participants 1 to 4 respectively) (Fig 7A). In Fig 7, we use the term ‘other’ to refer to the actions selected after an error at a leaf node that were neither *on-* nor *off-path*. They occur in the model because a node cannot be blamed for an error twice unless new evidence is collected from it. These actions are always directed at other leaf nodes. Intriguingly, participants also made a small fraction of actions that were neither *off-path* nor *on-path*, and most of them (72%) were also directed at leaf nodes (Fig 7A).

The heuristic model also reproduced other aspects of the participants’ behavior. The average number of points earned per trial was similar for model and data, even though the average reward was not used for model fitting (Fig 6A). Model and data also made a similar number of queries per level, and made approximately the same number of errors per trial at leaf nodes (Fig 6B).

The heuristic model allows us to distinguish between errors of perception and errors of strategy. As can be seen in Fig 6A, the average reward was positive for all subjects except for subject number 3. This is intriguing because this participant had better sensitivity to motion discrimination than subjects 2 and 4, as can be seen by comparing the signal-to-noise parameter of the model (*κ*, S1 Table). This dissociation between *κ* and the average reward obtained in the experiment is explained by differences in strategy. Participant 3 is the one who showed the highest proportion of *on-path* queries (Fig 7A, right column). *On-path* queries proved to be a strategic mistake. We could verify this with a model identical to the heuristic model except that it was fit to maximize reward. In this model, all the queries made after an error were off-path (Fig 7C). This predicts that participants would have achieved higher average reward if they would have made all the queries off-path, assigning the blame for the error to the decision that was made with the least confidence out of those in the error path.

The heuristic model also reproduced the participants’ behavior when analyzing separately the actions selected before and after an error at a leaf node (Fig 8). As observed in the data, in the heuristic model the action that follows an error can target different levels of the decision tree. After querying the internal nodes, transitions are often to the same level or to the level immediately below it. This is observed both before and after an error at a leaf node.

In the model, what determines whether an *off-path* or *on-path* query is made after an error at a leaf node is whether two or more decisions from the error-path were made with low confidence. Because low-confidence decisions are more frequent when motion is weak, the motion strength at the nodes not blamed for the error should be lower when participants made an *on-path* query than when they made an *off-path* query. We tested this prediction using logistic regression. If *s*_*n*1_ and *s*_*n*2_ are the motion strengths from the two nodes of the error-path that were not responsible for the error, the product of both, *s*_*n*1_ ⋅ *s*_*n*2_, should be lower when the participants did an *on-path* query instead of an *off-path* query after an error. This prediction is verified in the data (S6 Fig) (Eq 8, *β*_2_ = −4.9 ± 0.9, *p* < 10^−7^), and provides further evidence in favor of the mechanism we incorporated in the heuristic model to arbitrate between *off-path* and *on-path* queries.

### Learning

Although the analyzes and models that we presented assume that behavior is stable over trials, given that the participants carried out multiple, long experimental sessions spread over several days, it is likely that the decision strategies varied to some extent during the course of the experiment.

We analyzed how different aspects of the decision strategy changed with experience. The average reward and the number of re-queries increased gradually during the first ∼4–7 blocks (of 50 trials each) (S7(A) Fig). Importantly, even on the first block of trials, participants made more re-queries at the higher levels than at the lower levels of the decision tree (Eq 7, *β*_2_ = −0.9 ± 0.14, *p* < 10^−8^), an effect which was amplified in later blocks (S7(A) Fig). An analysis similar to that of Fig 8 conducted independently for the first and last 6 blocks of trials showed similar transition probabilities between levels for these two sets of trials (S7(B) Fig). Although a detailed analysis of the learning dynamics in our task is outside the scope of the current study, the analysis suggests that hallmarks of the heuristic strategy are already present in the first blocks of trials.

## Discussion

Adaptive behavior requires making accurate decisions, but also knowing what decisions are worth making. To study how people decide what to decide on, we investigated a novel task in which people had to find a target, hidden at the lowest level of a decision tree, by gathering stochastic information from the internal nodes of the decision tree. Our central finding is that a small number of heuristics explain the participants’ behavior in this complex decision-making task. The study extends the perceptual decision framework to more complex decisions that comprise a hierarchy of sub-decisions of varying levels of difficulty, and where the decision maker has to actively decide which decision to address at any given time.

Our task can be conceived as a sequence of binary decisions, or as one decision with eight alternatives. Participants’ behavior supports the former interpretation. Participants often performed multiple queries on the same node before descending levels, and they rarely made a transition from an internal node to a higher-level one before reaching a leaf node. This indicates that participants made categorical decisions about the direction of motion at the visited nodes before they decided to descend levels. This bias toward resolving uncertainty locally was not observed in an approximately optimal policy (Fig 8), and thus may reflect more general cognitive constraints that limit participants’ performance in our task [37]. A strong candidate is the limited capacity of working memory [38]. By reaching a categorical decision at each internal node, participants avoid the need to operate with full probability distributions over all task-relevant variables, favoring instead a strategy in which only the confidence about the motion choices is carried forward to inform future choices [39].

Participants often made many successive queries at an internal node. The number of queries was not predetermined, but depended on the difficulty of the decision and the depth of the decision in the decision tree. Participants requested more information when decisions were difficult [40–42]. They also made more re-queries at the higher levels of the decision tree than at the lower levels, which explains why the former decisions were more accurate (Fig 3). This strategy may seem sensible since misjudgments made at the higher level of the decision tree can cause the decision maker to spend a long time exploring the wrong branch of the decision tree. As stated by John von Neumann [43], errors add-up during long calculations and those committed early in the calculation are amplified at later portions of it. Surprisingly, however, the solution of an optimal model—in which we modified the payoffs to encourage multiple successive queries at the same node—shows that it would be more convenient to make more re-queries at the lower levels of the decision tree, unlike what was found in the data. The optimal strategy can be understood as follows. The optimal number of queries at a node depends on the balance between the cost of sampling and the benefit derived from the increase in expected accuracy. Since the decision maker does not know at the beginning of the trial the difficulty of the decisions that will be encountered in the future, sampling the root node multiple times can be sub-optimal because subsequent decisions may be difficult and thus other strategies (like searching directly over the leaf nodes) may be more rewarding. However, upon reaching the lower levels of the decision tree, a Bayesian decision maker can use the certainty in the decisions made at the higher levels to better assess the benefit of repeatedly sampling a low-level node, leading to an optimal strategy in which more queries are made at the lower levels of the decision tree.

An unexpected aspect of our results is that people did not accumulate motion information across re-queries. Instead, evidence was discarded if deemed unreliable to make a categorical motion choice. This conclusion—which contrasts with widely spread assumptions about the decision process in simple perceptual and value-based decisions—is based on a model-free analysis of how fluctuations in the stimulus motion information affect motion choices and on a formal comparison between models with and without integration. Using a model-free analysis similar to ours, Kiani et al. [30] showed that when participants are presented with two motion pulses separated by a brief interval, both pulses contribute almost equally to the motion choice. This result contrasts with ours in which only the second pulse had a significant effect on the ultimate choice. In our experiment we do not expect all pulses to have the same influence on motion choices even if the integration were perfect, because when the participant decided to consult a node again, it is likely that the evidence presented in the previous queries was weak—otherwise, the participant would not have made a re-query. With simulations, we verified that if a motion choice is made after two queries, the regression coefficient for the first motion pulse should be significantly different from zero. In contrast, the data—and simulations of the detection model—show no significant effect of the first pulse when a motion choice was made after two successive queries. Taken together, the analyses support a model in which only the last query informs the motion choice.

What explains the absence of integration in our task? One possibility is that participants discarded information that they would have used if the did not have the possibility of making a re-query. Alternatively, integration-free strategies may be more widespread than previously acknowledged. Many perceptual and value-based decisions could be based on a single, highly informative sample of evidence, but since experimenters do not know when this sample occurred, reverse correlation analyses may lead to the conclusion that decision makers accumulated information over longer periods of time than they actually did. While it may seem that these two alternative mechanisms (evidence integration and extrema detection) should lead to large differences in accuracy and response times, this is true only if the signal-to-noise ratio were known (including the noise in the stimulus and in the brain), which is usually not the case. If signal-to-noise is considered a free parameter that is fit to data, both models are indistinguishable for many of the tasks commonly used in the perceptual decision literature, including the random-dot motion discrimination task [26, 44–46].

Our results contrast with those that describe human decision making in terms of Bayesian inference. We did not find evidence that participants represent a joint probability distribution over all the decision-relevant variables. The Bayesian model, which does, yielded a performance far superior to that observed experimentally and displayed qualitatively different behavior. Because our task is more complex than those normally used in the perceptual decision making literature (e.g., [47, 48]), it could be that people deviate from optimal behavior as the decision problem becomes more complex. Alternatively, it is also possible that Bayesian and non-Bayesian models behave similarly if task complexity is low [26, 49, 50], and that more complex decisions are needed to reliably disambiguate between them.

Unlike the Bayesian model, which adapts its decision policy to any change in the reward structure, the heuristic model is limited to the space of decision policies that can be derived by modifying its parameters, such as the decision criteria and the ratio between on-path and off-path queries. While less flexible than the Bayesian model, the heuristic model is more flexible than the sampling norms commonly used in the literature (which we also explored in this study) such as probability-gain and information-gain, that do not have free parameters that can be adjusted to changes in the reward structure. Furthermore, it has been reported that people’s planning strategies are relatively insensitive to changes in the reward contingencies [51], and the tendency of people (and monkeys) to seek information even when it is detrimental to task performance can be interpreted as relative lack of sensitivity to reward information [34, 52]. A variant of our experiment in which rewards vary across blocks should be able to address the question of how sensitive people’s planning strategies are to the reward contingencies.

Our data show that the confidence in the accuracy of the motion decision–or other graded expectation of potential outcome–is carried forward in time to affect subsequent decisions. Confidence, however, is not enough to make optimal decisions in our task. For example, the confidence in a motion choice may be low because not enough information has been gathered, or because the evidence obtained was weak. The best subsequent action may be different in both cases: in the first case, it may be convenient to query the node again, while in the second it may be convenient to not query it again in the trial because it can be inferred from past queries that the source of evidence is unreliable. To distinguish between these strategies, it would be necessary to carry forward other measures of uncertainty in addition to confidence (e.g., confidence about confidence).

In the detection model, re-query decisions are made by comparing the strength of sensory evidence against a criterion. Identical results would be obtained if the criterion were set on confidence instead of evidence strength because there is a monotonic relation between the two. The placement of the criterion depends on tree level, which explains why performance differs across tree levels. The criterion also depends on the number of times a node was sampled in a row. Collapsing the criterion with the number of samples is a sensible strategy when the difficulty of the decision is unknown a priori: if many samples were obtained from a node and a decision has not yet been made, it is increasingly likely that the quality of the evidence is low and thus it may be convenient to hasten the decision by collapsing the criterion. A similar rationale justifies the use of collapsing decision termination bounds in reaction-time experiments [27, 31].

Confidence also informed the solution to the causal inference problem introduced by the ambiguous feedback delivered after an error. Participants’ behavior could be explained by a model that compares the confidence of the decisions made along the error-path to determine which decision is blamed for the error. If only one of decision was made with low confidence, the decision maker assigned the responsibility for the error to that node; if at least two decisions were made with low confidence, the highest-level node among them is queried again. Previous studies in humans and monkeys showed that confidence is used to decide whether the absence of reward was due to a perception error, or to a covert change in the stimulus-response contingency rules [21, 22]. Our results extend the role of confidence in disambiguating the cause of an error to the case of active sampling in decision hierarchies.

In this study, we defined confidence as the posterior probability that a motion choice is correct, calculated with Bayes rule. The posterior probability has proven to be a good proxy for confidence (up to a monotonic transformation) in previous studies using the random-dot motion task [20, 32, 33, 53, 54]. It is likely, however, that the posterior probability is only a first-degree approximation to people’s sense of confidence. The high degree of individual variability in the confidence reports [55, 56], the influence of non-task factors such as mood or personality traits [56, 57], experiments with subtle stimulus manipulations [58, 59] and modeling studies [60] suggest that there may be systematic deviations between confidence and the posterior probability that a choice is correct. It remains to be determined if some of the nuances in the computation of confidence, such as positive-evidence bias for confidence [58] or confidence’s insensitivity to evidence variability [61], influence people’s planning behavior in our task, and specially the assignment of blame after an error at a leaf node.

There are many differences between our study and previous planning studies in humans. Most of these studies relied on tasks without uncertainty (Classical planning) or tasks in which the uncertainty is limited to stochastic transitions between states (Markov Decision Processes, MDPs), and have focused on how people cope with the combinatorial explosion that occurs as the planning horizon increases [62–64], the depth with which people plan [65, 66], or the extent to which people use model-based or model-free strategies when learning from reinforcement [62, 67, 68]. The present study is different because we focus on how people disambiguate a single hidden state from a sequence of information-seeking and reward-seeking actions. The task belongs to the class of Partially-observable MDPs, in which an observation model is needed to make inferences about the current state from noisy or ambiguous observations. In this regard, our task is more similar to those used in the active sensing literature, which focuses on how the senses are directed to extract decision relevant information (see [69] for a review). Our study can be interpreted as extending the applicability of active sensing to tasks comprising a hierarchy of sub-decisions of varying levels of reliability.

While a detailed analysis of learning in our task is beyond the scope of this study, an analysis of how behavior changes with experience suggests that the basis of the heuristic strategy is already established early on. This implies that participants either automatically derived the heuristic strategy from the verbal description of the task, or learned it rapidly during the first few trials. Experience in the task may then be used to fine-tune the parameters of this initial strategy, rather than learning one ‘from scratch’. In this regard, the literature on program induction and meta-learning (e.g., [70, 71]) may be more relevant to understand how people derive appropriate control in our task than the literature on reinforcement learning that has been so influential in the study of human planning.

Also relevant are the studies that presented monkeys or humans with decision trees with stochastic information, in which the participants had simultaneous access to the evidence from the different bifurcations [19, 72–76]. These studies (which did not have an active sampling component) showed that lower-level decisions influence the decisions made at higher levels, suggesting that the evidence from the various bifurcations is combined before reaching a categorical decision for any one of them. An exception to the simultaneous presentation of evidence from all bifurcations is the study by Van den Berg et al. [20] in which the evidence for level-2 decision was presented only after a categorical decision was made at the root node. Although this task did not have planning, replanning or active sensing components, the study showed that level-2 decisions were made more quickly than level-1 decisions, consistent with our observation that the criterion Φ_*ℓ*_ becomes more liberal for decisions deeper in the decision tree.

With our task, we intended to strike a balance between the highly simplified decision-making tasks commonly used in systems neuroscience and the highly complex tasks used in psychology and cognitive sciences. Adapting our task to be performed by non-human primates may help elucidate the neurophysiological bases of active information sampling, heuristic planning and belief propagation, complementing recent studies in non-human primates trained in highly complex decision-making tasks (e.g., [77, 78]). Another potentially fruitful direction for future research is to adapt the paradigm present here for massive online studies. In addition to confirming the results presented in this study, based on a small number of highly experienced participants, it will allow us to explore how people’s decision strategies change with task variables such as the depth of the decision tree, the prior probability of different alternatives and the reward structure, and with variables external to the task such as trait measures.

Although our task was designed as a model of reasoning, many differences remain between our task and those decisions in which the evidence for the different sub-decisions is generated internally. An obvious difference is that while in our task the evidence for the different sub-decisions can only be evaluated one at a time, the brain can represent many streams of evidence simultaneously [79]. This difference may be less relevant that it may seem, because there is behavioral and neuroscientific evidence that even though the brain is capable of processing multiple streams of evidence in parallel, only one of them can be used to update a decision variable at each moment [39, 80, 81]. Therefore, the need to decide which memory, feeling or thought to use to inform a decision may be subject to the same type of arbitration rules that we have identified here. More fundamental differences are that the number of possible sources of evidence bearing on a decision can be vast [82, 83], that the hypothesis space may need to be expanded during the decision process [84, 85], and that richer internal models need to be queried to relate individual evaluations to the agent’s goals [86]. It might be fruitful to extend the paradigm presented here to approximate these more complex aspects of reasoning.

## Methods

### Ethics statement

The study was conducted at Columbia University (New York). All participants provided written informed consent. The study was approved by the Institutional Review Board of Columbia University Medical Center.

### Participants

Four participants (1 male and 3 female) took part in the study. All had normal vision and were naive about the purpose of the experiment. Three of the four participants took part in a previous study [33].

### Apparatus

Visual stimuli were presented on a CRT monitor with a screen refresh rate of 75Hz. A headrest and chin rest were used, and the eye position was monitored at 2,000 Hz using an Eyelink 1000 eye-tracker (SR Research Ltd., Mississauga, Ontario, Canada). The experiment was programmed in Matlab, using the Psychtoolbox library [87–89].

### Experimental design

Each participant completed the experiment over 5 to 8 sessions, of approximately 1 hour each. Participants completed 2–3 sessions per week, no more than one session per day. In a typical session, they completed 3–5 blocks of 50 trials each. In total, participants 1 to 4 completed 1450, 700, 1050 and 975 trials, respectively. This corresponds to a total 7015, 3921, 7817 and 5304 random-dot motion presentations per participant.

### Random-dot motion stimulus

The random dot motion stimulus was generated following methods described previously [48]. Each video frame shows one of three interleaved sets of dots. When replotted 40 ms later, each dot could be displaced by ±Δ*x* from its previous position, or it could be redrawn at a random location. The probability that a dot was redrawn in the direction of motion was equal to the motion strength. The displacement Δ*x* was such that the apparent speed of motion was 5 degrees of visual angle per second. The sign of the displacement was determined the direction of motion set for that node and trial. The dot density was 16.7/deg^2^/*s*, and the dots were visible within a circular aperture of 4 degrees of visual angle.

The motion strength and direction were assigned randomly and independently to each node at the beginning of the trial, and they remained constant throughout the trial. The motion strength assigned to each node was sampled from the list [3.2,6.4,12.8,25.6,51.2] %. Participants were presented with 227 ms of random-dot motion (17 video frames) every time they queried an internal node. Each random-dot motion movie was instantiated with a different random seed, so if participants queried the same node multiple times in a trial they were shown a different movie each time, albeit of the same motion strength and direction.

### Task description

The sequence of events within a trial is shown in S1 Movie. The decision tree (spanning 28° width and 14° height of visual angle) was presented at the beginning of the trial, including the 15 nodes and lines that connect them. When the participant’s gaze was within 2.2° of one of the nodes, it changed colors from red to pink. If then the participant pressed the keyboard’s space-bar, he or she was presented with a short pulse (227 ms) of random-dot motion. Finding the target finished the trial and delivered a positive reward. Selecting any of the other 7 leaf nodes triggered a low-pitched sound that indicated to the participants that the chosen leaf node was not the target.

Participants were informed that querying an internal node led to a loss of 1 point; selecting the wrong leaf node led to a loss of 3 points; and finding the target delivered a positive reward of 10 points. In all trials the participants found (eventually) the target, since it was the only way to end the trial and move on to the next one. After each trial, participants received feedback on how many points they scored in that trial, broken down by points lost for queries, points lost for selecting the wrong leaf node, and points earned for finding the target (see S1 Movie). At the end of each block, participants were shown a bar graph with the total number of points earned in the block and in all previous blocks.

### Data analysis

We used logistic regression to evaluate the influence of motion strength and tree level on the choices made after querying internal and leaf nodes. We used likelihood-ratio tests for nested models to evaluate the null hypothesis that one or more of the regression coefficients were equal to zero. In all regression models we incorporate the data from the different participants using indicator variables.

The logistic regression model used to determine the influence of motion strength and tree level on the motion choices is:

$$\begin{array}{c} \text{logit}\left[p_{\text{rightward}}\right]=\beta_{0}+\beta_{1}s+\beta_{2}s\ell +\sum_{i=1}^{N_{\text{subj}}-1}\beta_{2+i}I_{\text{subj}}\;, \end{array}$$ (6)

where *p*_rightward_ is the probability of a rightward motion choice (i.e., descend a level through the right branch), *s* is signed stimulus strength (positive for rightward motion, negative for leftward motion), *ℓ* is the level of the decision in the decision tree (with values 1 to 3 from the top down) and *I*_subj_ is an indicator variable that takes a value of 1 if the trial was completed by subject subj and 0 otherwise. For this analysis we only included those queries that were directly followed by a query at one of the two child nodes, which allow us to asses if the motion choice was correct or incorrect.

We used logistic regression to evaluate if the probability of querying the same internal node again (*p*_requery_) depended on motion strength and tree level:

$$\begin{array}{c} \text{logit}\left[p_{\text{requery}}\right]=\beta_{0}+\beta_{1}c+\beta_{2}\ell +\sum_{i=1}^{N_{\text{subj}}-1}\beta_{2+i}I_{\text{subj}}\;, \end{array}$$ (7)

where *c* is the unsigned motion strength. For this analysis we included all queries that were followed by the query of one of the two child nodes, or a re-query. As mentioned, these three type of choices correspond to the vast majority of the actions that follow the query of an internal node. The same regression model (using data from the first block of trials only) was used to evaluate whether tree level had an influence on *p*_requery_ on the first block of trials completed by each participant.

To determine if the proportion of *on-path* queries after an error at a leaf node depends on the motion strength of the nodes that were not blamed for the error, we used the following logistic regression model:

$$\begin{array}{c} \text{logit}\left[p_{on}\right]=\beta_{0}+\beta_{1}c_{b}+\beta_{2}c_{n,1}c_{n,2}+\sum_{i=1}^{N_{\text{subj}}-1}\beta_{2+i}I_{\text{subj}}\;, \end{array}$$ (8)

where *p*_*on*_ is the probability of doing an *on-path* query after an error at a leaf node, *c*_*b*_ is the motion strength of the node that was blamed for the error, and *c*_*n*,1_ and *c*_*n*,2_ are the motion strength at the other two nodes from the error-path. The heuristic model predicts that *β*_2_ should be negative since *on-path* queries are more likely when the decisions along the error path are made with low confidence, and low confidence choices are more frequent when motion is weak. For this analysis, we included those errors at leaf nodes for which we could classify the subsequent action as either an *on-path* or *off-path* query. Significance was evaluated with a likelihood ratio test for nested models with and without the *β*_2_ term.

We used the following logistic regression model to determine if the motion information in the stimulus had a significant leverage on the choice:

$$\begin{array}{c} \text{logit}\left[p_{\text{rightward}}\right]=\beta_{0}+\beta_{1}s+\beta_{2}\ell +\beta_{3}m+\sum_{i=1}^{N_{\text{subj}}-1}\beta_{3+i}I_{\text{subj}}\;, \end{array}$$ (9)

where *m* is the motion energy obtained from the motion stimulus. The motion energy is summed over time, thus it is only one value per query. The regression analysis includes those queries for which a rightward/leftward choice was made after exactly one query.

To determine if the motion energy from queries that were followed by a re-query had an influence on the ultimate choice, we used the following logistic regression model:

$$\begin{array}{c} \text{logit}\left[p_{\text{rightward}}\right]=\beta_{0}+\beta_{1}s+\beta_{2}\ell +\beta_{3}m_{\text{1st}}++\beta_{4}m_{\text{2nd}}+\sum_{i=1}^{N_{\text{subj}}-1}\beta_{4+i}I_{\text{subj}}\;. \end{array}$$ (10)

It is identical to the Eq 9 except that it includes two motion energy terms, *m*_1st_ and *m*_2nd_, which are the motion energies obtained from the two times that a node was queried before making a left/right motion choice. The analysis includes only those queries for which a rightward/leftward choice was made after exactly two queries.

In Fig 5 we show the time-course of the motion energy residuals. We calculate motion energy using previously published procedures [90, 91]. Briefly, the sequence of random dots presented on each trial was convolved with two pairs of spatiotemporal filters. Each pair is selective to one of the two directions of motion. Direction selectivity is achieved by addition and subtraction of the product of a temporal and a spatial filter. Opponent motion energy is computed by subtracting leftward from rightward preferring responses. After averaging over the two spatial dimensions, we obtain a time-varying signal which quantifies the fluctuations in motion energy during the course of the trial. The impulse response of the filters introduce a delay between the onset of the stimulus and response of the filters that is evident in Fig 5. The average motion energy is a linear function of motion strength. To calculate motion energy residuals, we subtract the expectation of the motion energy given by each node’s motion strength and direction.

The following logistic regression model was used to test whether the level of a node in the decision tree had an influence on the probability of being blamed for an error at a leaf node:

$$\begin{array}{c} \text{logit}\left[p_{\text{blame}}\right]=\beta_{0}+\beta_{1}c+\beta_{2}\ell +\sum_{i=1}^{N_{\text{subj}}-1}\beta_{2+i}I_{\text{subj}}\;, \end{array}$$ (11)

where *p*_blame_ is the probability that the node with motion strength *c* from level *ℓ* completed by subject *I*_subj_ is blamed for an error. In this analysis, each error at a leaf node contributes three entries to the logistic regression model, one for each node in the error path. The dependent variable takes a value of 1 for the node that was blamed for the error, and a value of 0 for the other two nodes. The analysis was performed independently for *on-path* queries, *off-path* queries, and the union of both, to estimate the influence of motion strength (*β*_1_ term) and tree level (*β*_2_) separately for these conditions.

In Fig 2 we show the conditional transition probabilities between pairs of states. For each subject *u*, *M*_*u*_(*i*, *j*) is the number of times that node *j* was queried after a query at node *i*. We calculate the conditional transition probabilities *W*_*u*_(*j*|*i*) diving each row in *M*_*u*_(*i*, *j*) by its sum. Then we compute the expectation of *W*_*u*_(*j*|*i*) over subjects, to obtain *W*(*j*|*i*). In the figures we only show the transitions for which *W*(*j*|*i*) is greater than 0.075. Line widths are proportional to *W*(*j*|*i*).

Fig 8 shows the (unconditional) transition probabilities between tree levels. For each subject *u*, we count the number of times that a node from level *ℓ*_*j*_ was queried after querying a node from level *ℓ*_*i*_, which is denoted by *N*_*u*_(*ℓ*_*i*_, *ℓ*_*j*_). To account for the difference in the number of trials across participants, we normalize *N*_*u*_(*ℓ*_*i*_, *ℓ*_*j*_) dividing it by the total number of queries (minus one) completed by each participant. Finally, we average across participants to obtain the normalized transition probabilities between levels, which we denote by *N*(*ℓ*_*i*_, *ℓ*_*j*_). The figure only shows the transitions for which *N*(*ℓ*_*i*_, *ℓ*_*j*_) is greater than 0.05. Line widths are proportional to *N*(*ℓ*_*i*_, *ℓ*_*j*_).

### Detection model

We assume that the momentary evidence comprises samples from a gaussian distribution and that the integration is unbounded for the duration of the stimulus. Therefore, the integral of the momentary evidence is also normally distributed. Following previous studies using random dot motion stimuli (e.g., [32]), the mean is assumed to be a linear function of motion strength,

$$\begin{array}{c} \mu_{c,d}=\kappa dct\;, \end{array}$$ (12)

where *κ* is the signal-to-noise, *d* indicates the net direction of motion (-1 for leftward and +1 for rightward motion) *c* is the motion strength and *t* = 0.227s is the stimulus duration. By convention, *μ* is positive for rightward motion, and negative for leftward motion.

The variance is also assumed to be a linear function of motion strength,

$$\begin{array}{c} \sigma_{c}^{2}=t\left(1+c\gamma \right)\;, \end{array}$$ (13)

which is known from previous studies to account for behavior better than a model with constant variance. It is a reasonable assumption as the variance of the motion energy derived from the stimuli increases with motion strength [33]. Slope *γ* is a free parameter of the model.

Each query of an internal node gives rise to a sample of motion evidence,

$$\begin{array}{c} e∼N(\mu_{c,d},\sigma_{c})\;, \end{array}$$ (14)

which depends on the node’s motion strength, *c*, and direction of motion, *d*.

The decision that is made after a query depends on the value of *e*. If *e* < −*ϕ*_*ℓ*_ or *e* > *ϕ*_*ℓ*_, the model considers that the direction of motion was left or right, respectively, and the next action is the selection of the corresponding child node. On the contrary, if |*e*| < *ϕ*_*ℓ*_, the evidence sample *e* is discarded and a new sample is obtained from the same node.

The criterion Φ_*ℓ*_ is a function of the level of the node in the decision tree, *ℓ*, and the number of previous queries made at the node, *n*_*q*_,

$$\begin{array}{c} \Phi_{\ell }=\phi_{\ell }(1+e^{-\lambda n_{q}})\;, \end{array}$$ (15)

where *ϕ*_*ℓ*_ is the base criterion which depends on level *ℓ*. Rate parameter λ controls how rapidly Φ decays with *n*_*q*_.

The detection model has 6 parameters in total: *θ* = {*κ*, *γ*, *ϕ*_1_, *ϕ*_2_, *ϕ*_3_, λ}. The model was fit the maximize the likelihood of the choices made after each query of an internal node,

$$\begin{array}{c} \hat{\theta }=\underset{\theta }{\text{argmax}}(\sum_{q=1}^{Q}\text{log}(P({\text{choice}}_{q}|c_{q},d_{q},\ell_{q},\theta )))\;, \end{array}$$ (16)

where choices can be +, − or *r* for choosing the right bifurcation, the left bifurcation, or doing a re-query, respectively. *c*_*q*_ and *d*_*q*_ are the motion strength and direction for query *q*, and *ℓ*_*q*_ is the level (1 to 3) of the node in the decision tree. We fit the model to the subset of decisions that were followed by a re-query or by a query at one of the two child nodes. As mentioned in Results, this includes the vast majority of decisions that follow the query on an internal node.

The behavior of the model is governed by the probability of exceeding the criteria at ±Φ_*ℓ*_:

$$p_{+}=\frac{1}{2}(1-\text{erfc}\;(\frac{\Phi_{\ell }-\mu }{\sqrt{2\sigma }}))$$ (17)

$$p_{-}=\frac{1}{2}(1-\text{erfc}\;(\frac{\Phi_{\ell }+\mu }{\sqrt{2\sigma }}))$$ (18)

$$p_{q}=1-p_{+}-p_{-}$$ (19)

where erfc is the complementary error function. The equations represent the probability mass that exists beyond or between ±Φ_*ℓ*_.

The model was fit independently for each participant using a Bayesian Optimization algorithm [92]. Solid lines in Figs 3 and 4 were generated with the best-fitting model for each participant. S1 Table shows the parameter values for the best-fitting model.

### Bayesian model

As mentioned in Results, the Bayesian model represents a probability distribution over the possible states *s* of the problem, P(s|E,V), conditioned on the motion samples obtained from past queries at the 7 internal nodes, *E*, and the set of leaf nodes that were already visited, *V*.

Before the first error at a leaf node (i.e., when *V* is the empty set), the distribution P(s|E,V) can be factorized as indicated in Eq 1. $P_{i}\left(c,d|E_{i}\right)$ in Eq 1 is the probability that the internal node *i* has motion strength *c* and motion direction *d*, given the set of motion samples *E*_*i*_ obtained from past queries at node *i*.

After querying node *i* and obtaining motion observation *e*, the posterior over motion direction at node *i* is updated following Bayes’ rule:

$$\begin{array}{c} P_{i}\left(c,d|E_{i}\right)≔\frac{P\left(e|c,d\right)P_{i}\left(c,d|E_{i}\right)}{\sum_{c,d}P\left(e|c,d\right)P_{i}\left(c,d|E_{i}\right)}\;, \end{array}$$ (20)

where P(e|c,d) is the likelihood function, examples of which are shown in Fig 4A. Before the first query, $P_{i}\left(c,d\right)$ is uniformly distributed.

The Bayesian model also represents the probability distribution B(T|E,V), which is the belief that the leaf node *T* is the target. If no error has yet been made at a leaf node (i.e., *V* is the empty set), the belief B(T|E,V) can be calculated from the distributions $P_{i}\left(d|E_{i}\right)$, which are easily obtained by marginalizing $P_{i}\left(c,d|E_{i}\right)$ over the motion strengths:

$$\begin{array}{c} P_{i}\left(d|E_{i}\right)=\sum_{c}P_{i}\left(c,d|E_{i}\right)\;. \end{array}$$ (21)

Then, the probability that leaf node *T* is the target can be factored as the product of $P_{i}\left(d|E_{i}\right)$ for the three nodes *i* that are in the path that connects the root node to *T* (see S2 Table). For example, the probability that target 10 is correct in Fig 1B is equal to the probability that the net direction of motion is rightward (*d* = 1) at nodes 1, 2 and 5. That is, $B\left(T=10\right)=P_{1}\left(d=1|E_{1}\right)\cdot P_{2}\left(d=1|E_{2}\right)\cdot P_{5}\left(d=1|E_{5}\right)$.

This factorization is no longer valid after an error at a leaf node, because the probabilities $P_{i}\left(d\right)$ are no longer independent when conditioned on the error. We use the following procedure to calculate the beliefs B(T|D,V) when the set *V* is not empty. First, we ignore the errors at the leaf nodes and use all the observations in *E* to calculate $P_{i}\left(d|E_{i}\right)$ for each internal node *i*, as previously described.

With $P_{i}\left(d|E_{i}\right)$, we calculate the probability of the *K* = 2^7^ possible combinations of motion directions at the 7 internal nodes. The examples shown in Fig 1B and 1C correspond to two such configurations. We use index *k* to identify a particular combination of motion directions at the internal nodes of the decision tree; for instance *k* = 0 could be used to identify the configuration in which the true direction of motion at every internal node is leftward. Ignoring the errors at leaf nodes, the probability of a particular combination *k* of motion directions at the 7 internal nodes is given by the product

$$\begin{array}{c} P_{k}=\prod_{i=1}^{7}P_{i}\left(d_{i}|E_{i}\right)\;, \end{array}$$ (22)

where *d*_*i*_ is 1 or -1 depending on whether, for combination *k*, the motion direction at node *i* is rightward or leftward, respectively.

We then incorporate the errors at the leaf nodes. We can zero the probability of all combinations *k* for which the correct leaf node is one of those that have already been visited and turned out not to be the target. For example, if leaf node 14 was queried and it was not the target, the 16 combinations of motion direction for which node 14 is the target can be discarded. For all combinations *k* that can be discarded by past errors at leaf nodes, we set the value of $P_{k}$ to zero, and renormalize such that the sum of the remaining $P_{k}$’s adds up to 1,

$$\begin{array}{c} P_{k}^{\text{norm}}=\{\begin{array}{cc} 0 & \text{if}\;T_{k}\in V\\ \frac{P_{k}}{\sum_{k^{\prime }}P_{k^{\prime }}1_{T_{k^{\prime }}\in V}} & \text{otherwise} \end{array}\;, \end{array}$$ (23)

where *T*_*k*′_ is the target for combination *k* and $1_{T_{k\prime }\in V}$ is an indicator variable that evaluates to 1 if the target for combination *k*′ is in the set *V* and to 0 otherwise.

Finally, we calculate the belief B(T|E,V) that leaf nodes *T* is the target. We do so for each leaf node *T* by adding the values of $P_{k}^{\text{norm}}$ over the 16 combinations *k* for which the correct leaf node is *T*,

$$\begin{array}{c} B\left(T|E,V\right)=\sum_{k}P_{k}^{\text{norm}}1_{T_{k}=T}\;, \end{array}$$ (24)

where $1_{T_{k}=T}$ is an indicator variable that evaluates to 1 if *T* is the target for combination *k*, and evaluates to zero otherwise.

So far we have explained how to update beliefs $P_{i}\left(c,d|E_{i}\right)$ and B(T|E,V) after a query at any of the 15 nodes. But we have not addressed how to select which node to query. For this, we used simulations.

Starting with each of the 15 possible actions, we evaluate the average cost that would be incurred until finding the target. Of course, the decision-maker does not know which leaf node is the target, so we implement the following procedure. The agent samples a state s* from the posterior distribution over states, P(s|E,V). Before an error at a leaf node, s* can be obtained by sampling the motion strength and direction for each internal node *i* from the distribution $P_{i}\left(c,d|E_{i}\right)$. To sample a state s* after an error at a leaf node, we first sample a combination of motion directions k* from the distribution $P_{k}^{\text{norm}}$, and then sample a motion strength for each node *i* from the distribution $P(c_{i}|d_{i})$, where *d*_*i*_ is the direction of motion at node *i* determined by the combination k*. The agent assumes that the state s* is the true state of the problem.

Then the agent simulates random actions until finding T*, the target corresponding to state s*. With probability $\frac{7}{15}$ the agent samples one of the internal nodes, chosen at random and with equal probability. With probability $\frac{8}{15}$, the agent samples one of the leaf nodes. In the latter case, the agent samples the leaf node that has the highest posterior probability of being the target. After each simulated action, the agent updates the distributions $P_{i}\left(c,d|E_{i}\right)$ and B(T|E,V) (Eqs 20 and 24). The observations *e*_*i*_ obtained after sampling an internal node are normally distributed with the parameters obtained from the fits of the detection model (Eq 14). The internal simulations of actions continues until finding the target T*. Once the target T* is found, the agent computes the cost incurred in finding it, using the true payoffs of the experiment. Note that this entire process occurs ‘in the head’ of the decision maker.

The process described above is repeated 2, 000 times starting with each of the 15 possible next actions, for a total of 15 × 2, 000 rollouts. To select the next best action, the agent averages the cost incurred in finding the target from each of the 15 possible next actions. The action chosen is the one for which the expected cost is the lowest. After executing the chosen action, an observation is obtained from the environment and used to update beliefs $P_{i}\left(c,d|E_{i}\right)$ and B(T|E,V). If the chosen node is not the target, the Bayesian decision-maker selects the next action repeating the simulation-based procedure just described.

### Heuristic model

The heuristic model extends the detection model with a mechanism to select which node to query after an error. The expected accuracy of the motion choices made along the error-path (or choice confidence) plays a key role in this process.

The confidence in having made a correct choice at internal node *i* is calculated using Bayes rule based on the sample of motion evidence *e* obtained the last time that node *i* was queried. The confidence in that rightward is the correct direction of motion is:

$$\begin{array}{c} {\text{conf}}_{i}^{+}=P_{i}\left(d=1|e\right)=\sum_{c}P_{i}\left(c,d=1|e\right)=\sum_{c}\frac{P\left(e|c,d=1\right)P_{i}\left(c,d=1\right)}{\sum_{d^{\prime }}\sum_{c^{\prime }}P\left(e|c^{\prime },d^{\prime }\right)P_{i}\left(c^{\prime },d^{\prime }\right)}\;, \end{array}$$ (25)

and confidence in a leftward choice is simply ${\text{conf}}_{i}^{-}=1-{\text{conf}}_{i}^{+}$.

The likelihood P(e|c,d=1) is given by the probability density function of the normal distribution (Eq 14). If many successive queries were made at an internal node, confidence is based on the motion evidence from the last query. This assumption was adopted for consistency with the detection model. After an error at a leaf node, the decision maker compares confidence for the three decisions in the error path to determine the best next action. Whether we use the confidence in a rightward or leftward choice depends on the directions of motion required to reach the leaf node where the error was made. For instance, if an error was made at node 12 in Fig 1B, then the confidence that we use for the decisions made at nodes 1, 3 and 6 is the confidence in a leftward choice, because all these choices had to be leftward for node 12 to be the target.

We incorporated two additional assumptions in the heuristic model. One is that once an internal node has been blamed for an error, it cannot be blamed again until a new sample is obtained from it. Without this assumption the decision maker can blame the same node repeatedly when only one of the decisions in the error-path was made with low confidence. Because of this assumption, sometimes none of the nodes in path ***P***_***T***_ can be held responsible for the error. In this case the decision maker queries a leaf node at random and with equal probability from among those not yet visited. Another modeling assumption is that the decision maker can remember the leaf nodes already visited and thus does not query the same leaf node twice in the same trial. If the model makes a motion choice at level 3 of the decision tree that would lead to a leaf node that has already been visited in the trial, then the decision maker selects the next action as if it had selected that leaf node and obtained negative feedback, without having to query the leaf node again.

The only parameter of the heuristic model (*ω*) was fit to minimize the difference between the proportion of *on-path* queries between model and data,

$$\begin{array}{c} err=|p_{\text{on-path}}^{\text{model}}-p_{\text{on-path}}^{\text{data}}|\;, \end{array}$$ (26)

where *p*_on-path_ is the number of *on-path* queries divided by the total number of errors at leaf nodes.

The results of heuristic model are based on 50,000 simulated trials per participant.

### Shallow sampling norms

The criteria used to select actions using shallow sampling norms were described in Results. The expectations in Eqs 2–4 are computed over the possible observations *o* than can follow action *a*, *p*(*o*|*a*, *E*, *V*), where *E* and *V* contain the set of motion samples and already visited leaf nodes, respectively.

If action *a* is the query of leaf node *T*, observation *o* can take two possible values corresponding to target reached and not-reached. In this case the likelihood P(o|a,E,V) is given by B(T|E,V) and (1-B(T|E,V)) for positive and negative feedback, respectively.

Instead, if action *a* is the query of internal node *i*, then the observation *o* is a sample of motion information, and computing its likelihood requires marginalizing over the possible values of motion strength *c* and direction *d*:

$$\begin{array}{c} P\left(o|a,E,V\right)=\int_{d}\int_{c}P\left(o|a,c,d,E,V\right)P_{i}\left(c,d|E,V\right)\;, \end{array}$$ (27)

where

$$\begin{array}{c} P_{i}\left(c,d|E,V\right)=P_{i}\left(c|d,E_{i}\right)P_{i}\left(d|E,V\right)\;. \end{array}$$ (28)

For the first term on the right side of Eq 28, the conditionalization on *E* and *V* can be simplified to *E*_*i*_ because once we condition on motion direction (*d*), the probability of the different motion strengths becomes independent of past errors and of the motion samples obtained from other nodes. $P_{i}(d|E,V)$ is calculated from the enumeration of the *k* possible combinations of motion direction at the internal nodes,

$$\begin{array}{c} P_{i}\left(d|E,V\right)=\sum_{k}P_{k}^{\text{norm}}1_{d_{i}^{k}=d}\;, \end{array}$$ (29)

where the sum is over all combinations *k* (as explained in the section on the Bayesian model) and $1_{d_{i}^{k}=d}$ is an indicator variable that evaluates to 1 if for combination *k* the direction of motion at internal node *i* is equal to *d* and evaluates to zero otherwise.

## Supporting information

S1 Table Best-fit parameters for the detection and heuristic models. (PDF)

S2 TableAll paths *P*_*T*_ from the root node to leaf node *T*.

$P_{T}^{\left(\ell \right)}$ represents the element of ***P***_***T***_ from level *ℓ*. Node-numbering follows the convention of Fig 1D.(PDF)

S1 Movie Hierarchical decision making task. Screen recording of three trials of the experiment. The cursor is used to simulate the participant’s gaze; it was not shown in the experiment.
(MP4)

S1 Fig Motion energy kernels calculated separately for the three levels of the decision tree. We analyze the left/right choices made after two successive queries of the same internal node. The upper and lower rows show the motion energy residuals obtained from the first and second queries, respectively, sorted by the left/right choice made after the second query. The decisions made at each level of the decision tree were analyzed separately, and are shown here in columns. Shading indicates s.e.m. A comparison of nested regression models favored the one without the motion information from the first motion pulse (Eq 10, Δ*BIC* = 1.3, 6.5 and 5.4 for levels 1–3 respectively, all supporting the model without the *β*_3_ term).
(PDF)

S2 Fig Statistical comparison to alternative models. Difference in Bayesian information criterion (BIC) between the detection model and five alternative models. Positive values indicate support for the detection model. From left to right, the five alternative models are: (1) a model in which the evidence from successive queries at an internal node are integrated, unlike the detection model in which only the last query influences the left/right choice; (2) a model in which there is a single common criterion *ϕ* for the three levels of the decision tree, but where the signal-to-noise ratio (*κ*) could take different values for the three levels of the decision tree; (3) similar to the previous model, except that *ϕ* could also take different values for each level of the decision tree, as in the detection model; (4) model identical to the detection model except that the noise was independent of motion strength (i.e., *γ* = 0); (5) model in which the criterion *ϕ* depends on *q*—the order of the query in the trial—parameterized as: *ϕ* = *ϕ*_∞_ + (*ϕ*_0_ − *ϕ*_∞_)*e*^*η*(*q*−1)^, where *η*, *ϕ*_0_ and *ϕ*_∞_ are fitted parameters.
(PDF)

S3 Fig The relation between tree level and the probability of making a re-query is not mediated by query number or elapsed time. Proportion of queries that were followed by a re-query, as a function of the order of the query (left panel), and the time of query in the trial (right panel). The proportion of re-queries was calculated separately for the three levels of the decision tree. Re-queries were more likely at higher levels of the decision tree, even for the same query number or elapsed time. This indicates that neither query number nor elapsed time can explain away the influence of tree level of the probability of a re-query. In the left panel, we only include those conditions with at least 6 queries from each participant. The data-points are averages across participants. Error bars represent s.e.m. across participants. In the right panel, we calculate the proportions in sliding windows of 300 queries each, after sorting the queries by elapsed time.
(PDF)

S4 Fig Behavior of the Bayesian model with less costly sampling. The Bayesian model was derived for the case in which the cost of querying the internal nodes of the decision tree was reduced to 30% of its true value. (A) Average number of queries per trial at levels 1–3 and average number of errors at leaf nodes. The data is shown in green (similar to Fig 6A), and the behavior of the Bayesian model with less-costly sampling is shown in orange. Error-bars indicate s.e.m. (B-E) Similar to Fig 2, but for the actions selected by the Bayesian model with less costly sampling. It shows the conditional transition probabilities from nodes of level 1–3 (panels B–D), and from the leaf nodes (panel E). The width of the red lines is proportional to the conditional transition probabilities between nodes.
(PDF)

S5 Fig Proportion of correct motion choices and re-queries for the Bayesian model with modified reward contingencies. Same as Fig 3, but the data-points were obtained from the Bayesian model with cheaper sampling (50% of its true value) and even cheaper re-queries (5%). Data-points are based on 2,000 simulated trials per participant. The solid lines are fits of a detection model similar to the one used in Fig 3. Unlike the data, the Bayesian model does more re-queries at the lowest level of the decision tree.
(PDF)

S6 Fig The proportion of *on-path* queries depends on the motion strength at the nodes not blamed for the error. The figure shows the proportion of *on-path* queries (sum of all *on-path* queries divided by the sum of all *on-path* and *off-path* queries) as a function of the product of the motion strength of the two nodes from the error-path that were not blamed for the error. The heuristic model predicts that there ought to be fewer *on-path* queries when the motion is stronger for the two nodes not blamed for the error. This prediction is verified in the data (see statistical analysis in the main text). The dashed line is the fit of an exponential function to individual-trial data. Error bars indicate s.e.m.
(PDF)

S7 Fig Dynamics of task performance. (A) Average reward residuals (top) and proportion of re-queries (bottom) as a function of block number. The reward residuals are obtained subtracting from the reward obtained on each trial, the expected reward given the trial’s motion strength at each internal node. The reward expectation was calculated with a linear regression model fit independently for each participant, using the motion strength at each of the 7 internal nodes (plus an intercept) as independent variables. The bottom panel shows the proportion of re-queries at each level of the decision tree, calculated from the subset of queries in which the query of an internal node was followed by a re-query or by the query of one of the two child nodes. Each block has 50 trials. Data are averages across participants. Error bars indicate s.e.m. across participants. (B) As Fig 8, but calculated independently for the first and last 6 blocks completed by each participant. Transition probabilities between levels are largely similar for the two sets of trials.
(PDF)
