# Supplementary material for: Decision prioritization and causal reasoning in decision hierarchies
Source: PLoS Comput Biol. 2021 Dec 31;17(12):e1009688. doi: 10.1371/journal.pcbi.1009688 (PMC8719712; doi:10.1371/journal.pcbi.1009688)
Supplement: S1 Table — (PDF) [file pcbi.1009688.s001.pdf]

Participant ID	κ	ϕ_1	ϕ_2	ϕ_3	γ	λ	ω
S1	18.33	0.35	0.2	0.09	2.13	0.13	0.8
S2	12.69	0.32	0.15	0.08	1.11	0.35	0.79
S3	13.81	0.45	0.35	0.28	3	0.2	0.85
S4	11.22	0.44	0.21	0.04	1.75	0.06	0.64
