# Supplementary material for: Decision prioritization and causal reasoning in decision hierarchies
Source: PLoS Comput Biol. 2021 Dec 31;17(12):e1009688. doi: 10.1371/journal.pcbi.1009688 (PMC8719712; doi:10.1371/journal.pcbi.1009688)
Supplement: S2 Table — PT(ℓ) represents the element of PT from level ℓ. Node-numbering follows the convention of Fig 1D. (PDF) [file pcbi.1009688.s002.pdf]

Leaf node (T)	$P_T^{(1)}$	$P_T^{(2)}$	$P_T^{(3)}$
8	1	2	4
9	1	2	4
10	1	2	5
11	1	2	5
12	1	3	6
13	1	3	6
14	1	3	7
15	1	3	7
