# Supplementary figures and images for: Decision prioritization and causal reasoning in decision hierarchies
Source: PLoS Comput Biol. 2021 Dec 31;17(12):e1009688. doi: 10.1371/journal.pcbi.1009688 (PMC8719712; doi:10.1371/journal.pcbi.1009688)

*First of  
two queries*

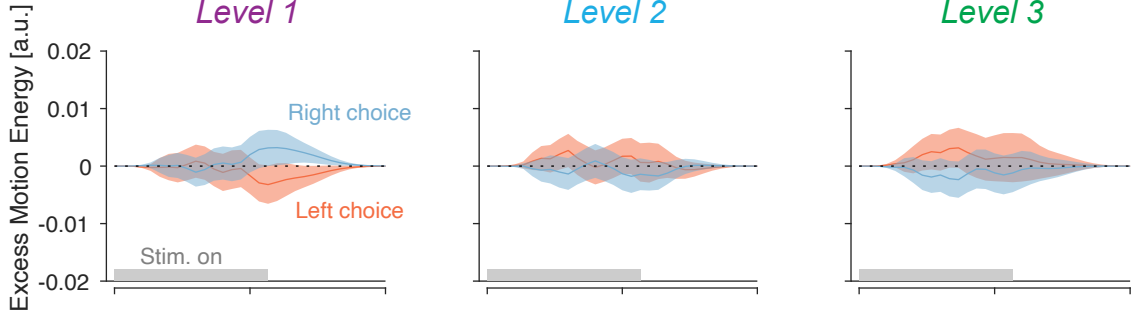

*Second of  
two queries*

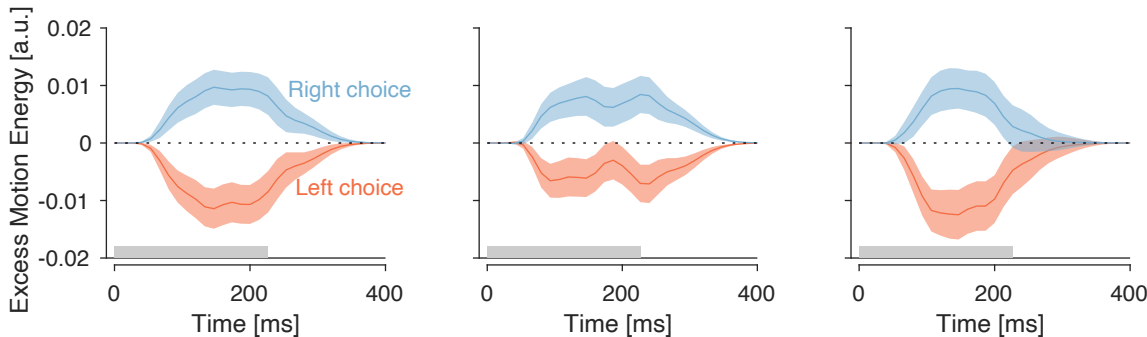

Supplement: S1 Fig — We analyze the left/right choices made after two successive queries of the same internal node. The upper and lower rows show the motion energy residuals obtained from the first and second queries, respectively, sorted by the left/right choice made after the second query. The decisions made at each level of the decision tree were analyzed separately, and are shown here in columns. Shading indicates s.e.m. A comparison of nested regression models favored the one without the motion information from the first motion pulse (Eq 10, ΔBIC = 1.3, 6.5 and 5.4 for levels 1–3 respectively, all supporting the model without the β3 term). (PDF) [file pcbi.1009688.s004.pdf]

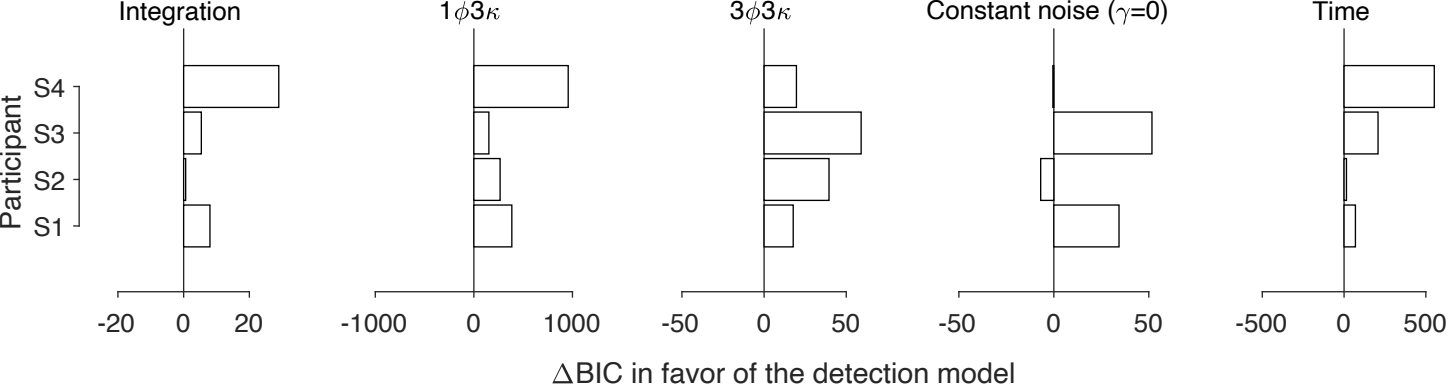

Supplement: S2 Fig — Difference in Bayesian information criterion (BIC) between the detection model and five alternative models. Positive values indicate support for the detection model. From left to right, the five alternative models are: (1) a model in which the evidence from successive queries at an internal node are integrated, unlike the detection model in which only the last query influences the left/right choice; (2) a model in which there is a single common criterion ϕ for the three levels of the decision tree, but where the signal-to-noise ratio (κ) could take different values for the three levels of the decision tree; (3) similar to the previous model, except that ϕ could also take different values for each level of the decision tree, as in the detection model; (4) model identical to the detection model except that the noise was independent of motion strength (i.e., γ = 0); (5) model in which the criterion ϕ depends on q—the order of the query in the trial—parameterized as: ϕ = ϕ∞ + (ϕ0 − ϕ∞)eη(q−1), where η, ϕ0 and ϕ∞ are fitted parameters. (PDF) [file pcbi.1009688.s005.pdf]

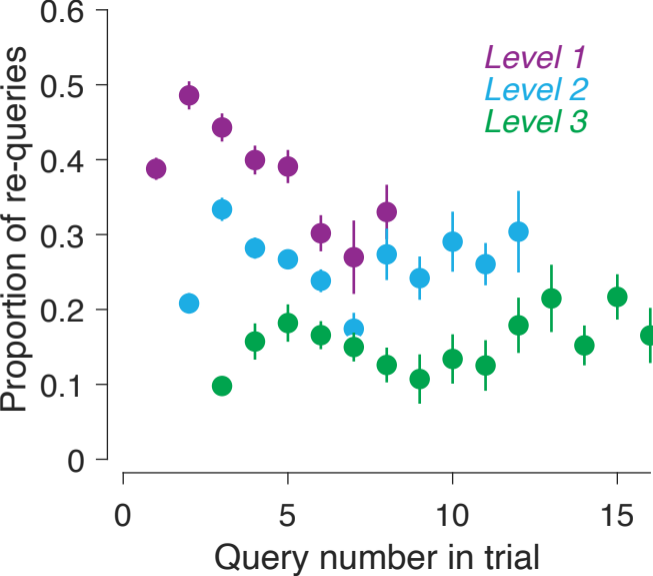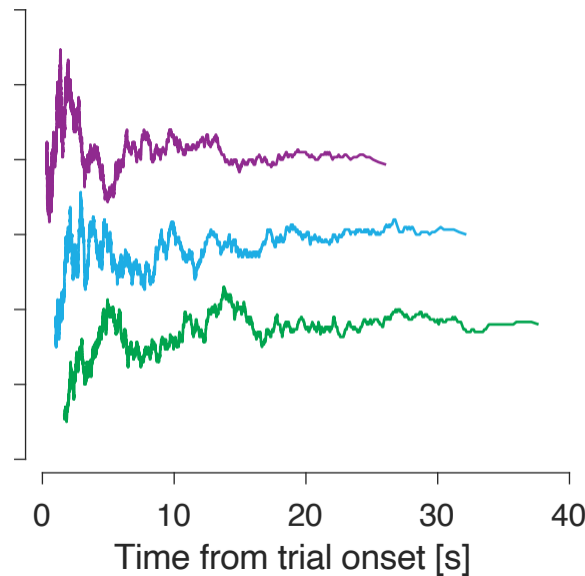

Supplement: S3 Fig — Proportion of queries that were followed by a re-query, as a function of the order of the query (left panel), and the time of query in the trial (right panel). The proportion of re-queries was calculated separately for the three levels of the decision tree. Re-queries were more likely at higher levels of the decision tree, even for the same query number or elapsed time. This indicates that neither query number nor elapsed time can explain away the influence of tree level of the probability of a re-query. In the left panel, we only include those conditions with at least 6 queries from each participant. The data-points are averages across participants. Error bars represent s.e.m. across participants. In the right panel, we calculate the proportions in sliding windows of 300 queries each, after sorting the queries by elapsed time. (PDF) [file pcbi.1009688.s006.pdf]

**A**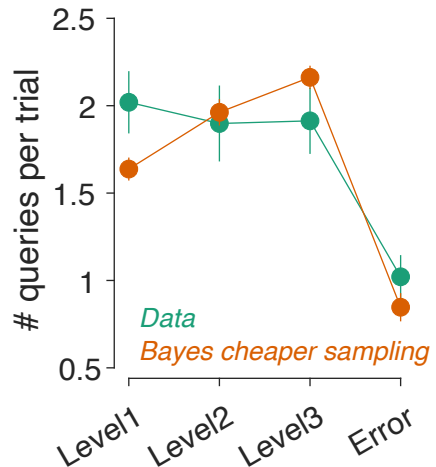**B**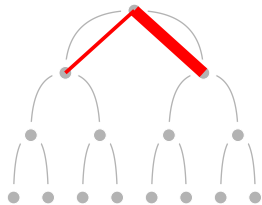**C**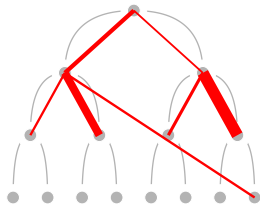**D**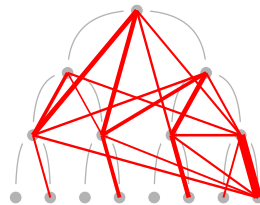**E**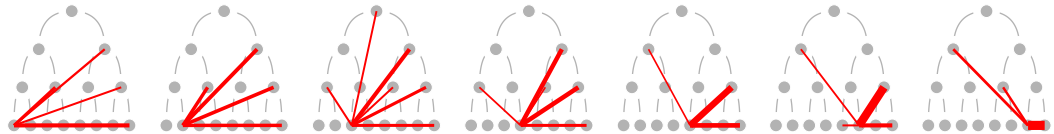

Supplement: S4 Fig — The Bayesian model was derived for the case in which the cost of querying the internal nodes of the decision tree was reduced to 30% of its true value. (A) Average number of queries per trial at levels 1–3 and average number of errors at leaf nodes. The data is shown in green (similar to Fig 6A), and the behavior of the Bayesian model with less-costly sampling is shown in orange. Error-bars indicate s.e.m. (B-E) Similar to Fig 2, but for the actions selected by the Bayesian model with less costly sampling. It shows the conditional transition probabilities from nodes of level 1–3 (panels B–D), and from the leaf nodes (panel E). The width of the red lines is proportional to the conditional transition probabilities between nodes. (PDF) [file pcbi.1009688.s007.pdf]

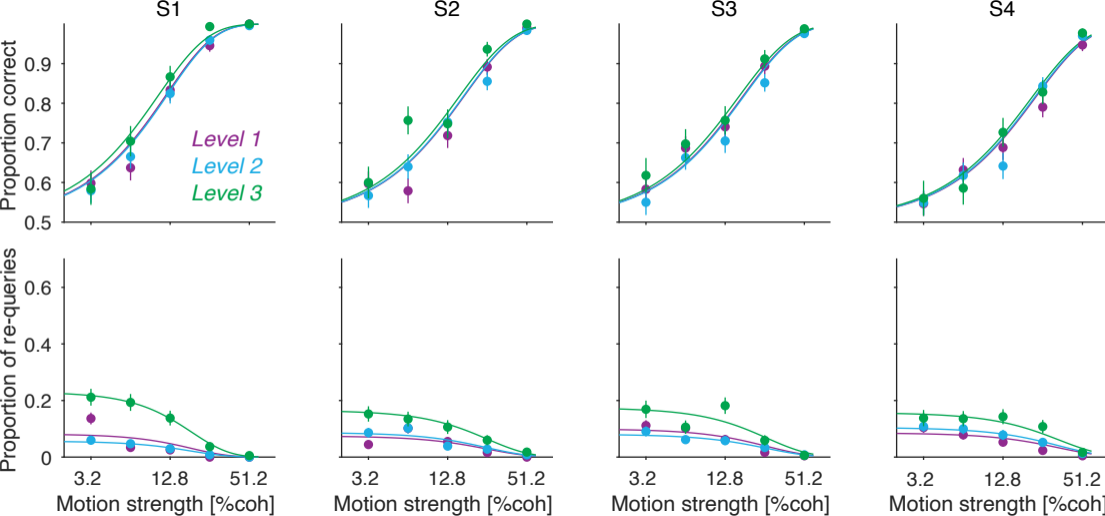

Supplement: S5 Fig — Same as Fig 3, but the data-points were obtained from the Bayesian model with cheaper sampling (50% of its true value) and even cheaper re-queries (5%). Data-points are based on 2,000 simulated trials per participant. The solid lines are fits of a detection model similar to the one used in Fig 3. Unlike the data, the Bayesian model does more re-queries at the lowest level of the decision tree. (PDF) [file pcbi.1009688.s008.pdf]

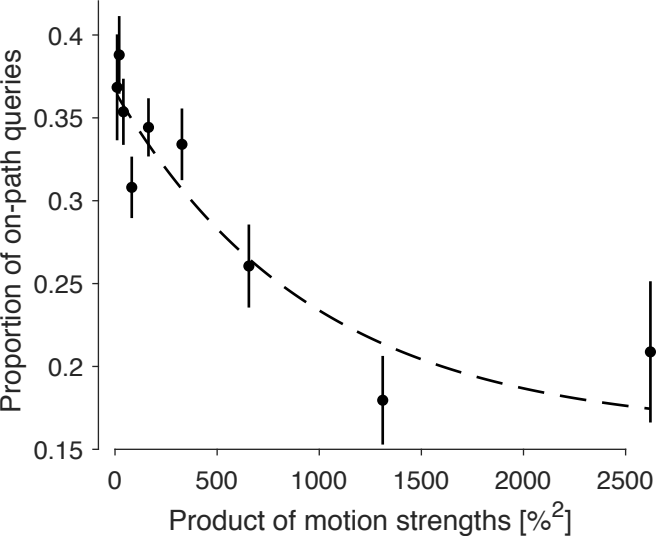

Supplement: S6 Fig — The figure shows the proportion of on-path queries (sum of all on-path queries divided by the sum of all on-path and off-path queries) as a function of the product of the motion strength of the two nodes from the error-path that were not blamed for the error. The heuristic model predicts that there ought to be fewer on-path queries when the motion is stronger for the two nodes not blamed for the error. This prediction is verified in the data (see statistical analysis in the main text). The dashed line is the fit of an exponential function to individual-trial data. Error bars indicate s.e.m. (PDF) [file pcbi.1009688.s009.pdf]

**A**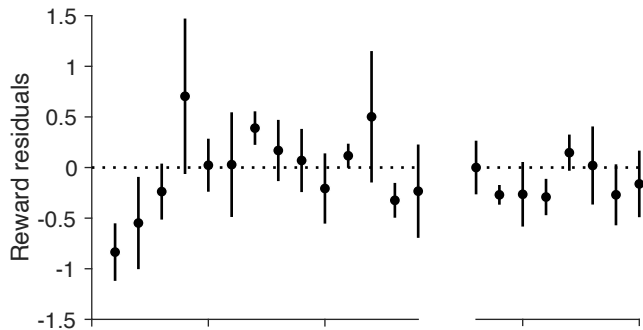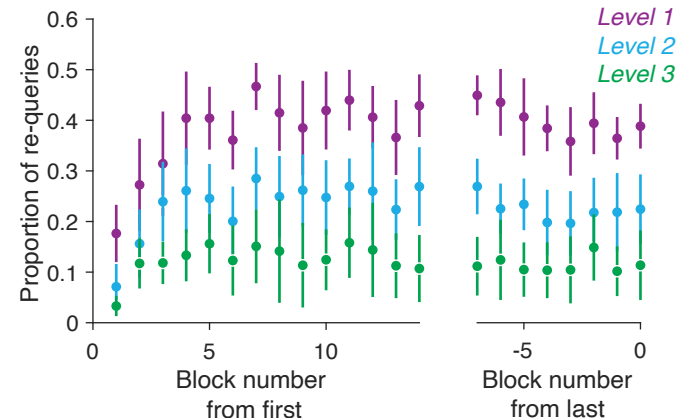**B**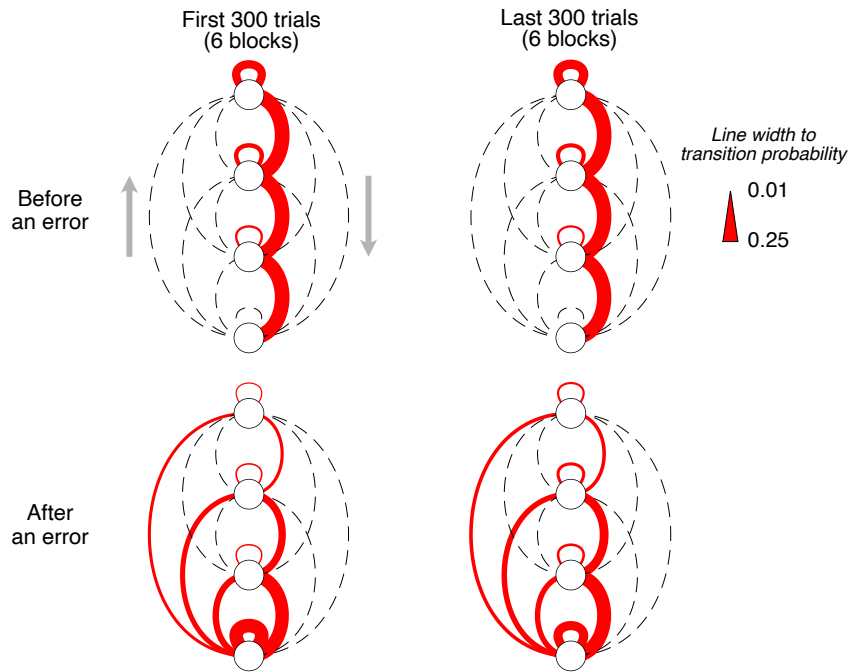

Supplement: S7 Fig — (A) Average reward residuals (top) and proportion of re-queries (bottom) as a function of block number. The reward residuals are obtained subtracting from the reward obtained on each trial, the expected reward given the trial’s motion strength at each internal node. The reward expectation was calculated with a linear regression model fit independently for each participant, using the motion strength at each of the 7 internal nodes (plus an intercept) as independent variables. The bottom panel shows the proportion of re-queries at each level of the decision tree, calculated from the subset of queries in which the query of an internal node was followed by a re-query or by the query of one of the two child nodes. Each block has 50 trials. Data are averages across participants. Error bars indicate s.e.m. across participants. (B) As Fig 8, but calculated independently for the first and last 6 blocks completed by each participant. Transition probabilities between levels are largely similar for the two sets of trials. (PDF) [file pcbi.1009688.s010.pdf]
